# Environmental Risk Assessment of Toxic Emissions into the Air Resulting from Waste Fires

**DOI:** 10.3390/toxics14100892

**Published:** 2026-10-06

**Authors:** Łukasz Szałata, Piotr Jadczyk, Maksym Byelyayev

**Affiliations:** 1 Department of Environmental Biology and Atmospheric Protection, Faculty of Environmental Engineering, Wroclaw University of Science and Technology, Pl. Grunwaldzki 9, 50-377 Wrocław, Poland; piotr.jadczyk@pwr.edu.pl; 2 Eko-Biegly Association, 50-155 Wroclaw, Poland; biuro@ekobiegly.pl

**Keywords:** environmental risk, waste incineration, air emissions

## Abstract

Uncontrolled waste fires pose a significant environmental and human health risk due to the emission and dispersion of hazardous gases and particulate matter. This study develops a receptor-oriented methodology for screening environmental risk based on human and environmental exposure to air pollutants released during waste storage facility fires. The approach combines Gaussian plume modelling (Pasquill stability classes) with a comparison of predicted pollutant concentrations against reference values established by Polish legislation, the US EPA, OSHA, and NDS standards. The methodology was applied to a case study involving the uncontrolled burning of waste from municipal waste treatment. For a waste mass of 14,000 kg, a fire duration of 3.5 h, and defined meteorological scenarios, concentration fields were modelled for selected pollutants, including PM10 and PM2.5, at receptor points and in the nearest residential area located approximately 700 m from the fire site. Under the most unfavourable analysed conditions (stability class 6, wind speed 1 m/s, southerly wind), the predicted concentrations at residential receptors remained below the applicable reference values. The results indicate that the proposed modelling framework can provide a rapid screening tool for environmental risk assessment following uncontrolled waste fires, particularly when direct emission or air-quality measurements are unavailable.

## 1. Introduction

In recent years, incidents of uncontrolled waste burning at waste collection sites have become a significant problem in Poland. Between 2017 and 2022, there were 754 such incidents (NIK report) [1]. Among the reasons cited for this phenomenon are the ban on waste imports into China—which in previous years had accepted significant quantities from more economically developed countries—and the ban on the landfilling of combustible waste introduced in Poland, coupled with a simultaneous increase in the requirements that customers place on alternative fuels [2,3]. Risks of this kind also arise as a result of the growing volume of waste batteries, which are increasingly ending up at waste management facilities that do not process batteries [4,5].

In Poland, such incidents are generally the subject of investigations by the public prosecutor’s office, which are usually conducted with a view to identifying an offence under the Criminal Code, that is, bringing about an incident which threatens the safety of many people or property on a large scale, and towards criminalising the unauthorised handling of substances and waste, which may endanger human life or health or cause a deterioration in the quality of water, air or land, or damage to the plant or animal world. 

Uncontrolled waste incineration produces toxic by-products, most of which are released into the atmosphere, they may therefore reduce air quality and pose a threat to the life and health of people breathing such air. When assessing whether these risks have materialised, difficulties may arise in determining the volume of harmful gases and pollutants to which people have been exposed, the duration and extent of that exposure, and identifying the specific levels of damage caused. The variation in emissions of harmful substances into the atmosphere results from the incineration of many different types of waste [6,7,8,9,10,11,12]. Information on the toxicity of pure combustion products is now considered standard knowledge [13]. Mixtures of products resulting from the combustion of certain types of waste were subjected to biotests, which revealed mutagenicity and cytotoxicity [9,14]. For genotoxic mutagens, a non-threshold model of action is traditionally assumed, according to which even a very small dose may carry a risk of inducing mutations. However, recent research suggests that threshold values may exist for certain mutagenic mechanisms [15]. This means that it is not possible to determine safe concentrations for them, below which they do not cause mutation. 

Illegal waste incineration is typically dispersed, long-term and difficult to accurately identify. Emissions occur without any control over the combustion parameters, which makes it impossible to determine precisely the type of waste being incinerated and the volume of emissions of individual pollutants [16]. This process encourages the formation of particulate matter, carbon monoxide, polycyclic aromatic hydrocarbons, dioxins, furans, volatile organic compounds and heavy metals. Furthermore, emissions may originate from multiple sources simultaneously, which makes it significantly more difficult to assess their impact on the environment and human health. A landfill fire, on the other hand, is a sudden and short-lived event that can cause very high instantaneous concentrations of pollutants. Its scale depends primarily on the area affected by the fire, the type and quantity of waste, and meteorological conditions. 

The first step in environmental risk assessment may be to determine the quantity of waste incinerated; the next involves establishing the quantity and composition of gases and particulate matter released into the atmosphere, followed by determining the dispersion range of these pollutants and the scale of their impact. The quantity of incinerated waste can usually be determined from records kept by a professional waste management facility or by calculating the volume of the storage site. Determining the qualitative and quantitative composition of emissions currently presents a challenge; the solution depends on establishing the actual composition of the waste itself, which undoubtedly has a significant influence on the quantity and quality of the resulting emissions. Research findings in this field show some variation, likely stemming from uncertainty regarding the compositional variability of the incinerated waste. Some researchers measure the quantities of carbon oxides (CO and CO_2_) generated by incinerating a unit mass of municipal waste [6], while others focus on the quantities of other pollutants produced during the process [7]. Some studies focus on the quantities of various combustion products derived from non-metallic automotive shredder residue; it can be assumed—as an approximation—that these results also apply to the incineration of plastics from other sources [8]. Lemieux and Ryan investigated combustion products per unit mass of car tires [9]. These studies enable the estimation of gas and particulate emission levels that could result from the uncontrolled incineration of the same or similar types of waste at other locations. The EPA presents a wide range of results regarding emission factors for the uncontrolled burning of waste in document AP-42 (fifth edition, Volume I, Chapter 2: Solid Waste Disposal) [17]. 

To determine the extent of air pollutant spread, mathematical dispersion models are used to calculate their dispersion. An air pollutant dispersion model is a tool (mathematical, physical, or computational) that, in this context, describes how air pollutants disperse in the environment [18]. It is used for air quality assessment, evaluating the impact of industrial installations and spatial planning. 

To determine the environmental risk associated with exposure to toxic gas and dust emissions, appropriate reference values are adopted. These values are established in accordance with legal regulations or based on literature data if regulations do not specify them. Risk assessment based on the toxicity of air emissions involves determining pollutant levels at locations where people are present during an incident and evaluating whether the pollutant concentration at a given location reaches a level that could pose a threat. When assessing potential harm to humans, values such as NDS, NDSCh, and NDSP as well as RfC and PEL-TWA are typically taken into account. 

## 2. Materials and Methods

### 2.1. Determination of Gas and Dust Emissions

Determining the quantity of gases and dust emitted into the atmosphere requires establishing the mass of incinerated waste and having data on the amounts of specific pollutants generated by the incineration of a unit quantity of waste. The quantities of pollutants generated from the combustion of a unit amount of cut and shredded car tires [9], non-metallic residues from vehicle shredding facilities [8], municipal waste [6,7], agricultural waste [10], and military waste [11,12] were determined. Uncontrolled waste combustion often occurs at temperatures significantly lower than those found in waste incineration plants. Temperatures during uncontrolled open-air waste combustion can range from 80 to 230 °C for surface burning and from 309 to 406 °C for full combustion. At the point of ignition and in the center of the combustion zone, the temperature is significantly higher, reaching 704 °C [19,20]. High temperatures are particularly characteristic of plastic waste combustion sites, especially where large quantities have accumulated and the materials are highly flammable [21]. Combustion temperature significantly influences the composition of combustion products; therefore, it is crucial that risk assessments regarding uncontrolled waste burning be based on emission data from similar incidents rather than on data obtained from combustion under optimal laboratory conditions.

This study analysed a case study as an example of a typical waste fire incident that took place in a selected town in south-western Poland. The quantity of incinerated waste was calculated based on records maintained by the facility, taking into account waste received and waste transferred for disposal as a result of the incident. The dispersion modelling was performed using meteorological data during the actual fire episode, including wind speed, wind direction, air temperature and atmospheric stability. Emission factor values were adopted based on data from the document “EPA AP-42, Vol. I, CH 2.5: Open Burning” [22]. Furthermore, data regarding the duration of emissions—i.e., the duration of the fire—were derived from information provided by the State Fire Service. The results of the emission calculations are presented below (Table 1).

The emission calculations presented in Table 1 are the product of the emission factor and the quantity of waste incinerated. For example, for benzene: the emission factor is 0.0123 mg/kg; 0.0123 mg/kg × 14,000 kg of waste = 172.2 mg (or 0.0001722 kg) of total benzene emissions from the incinerated waste. To calculate the hourly emission rate, the total emission value was divided by the duration of the waste incineration—specifically, 0.0001722 kg / 3.5 h = 0.0000492 kg/h of benzene emissions. According to data from the CEIDARS database, the PM10 fraction accounts for 7.3% of the total suspended particulate matter, while the share of the PM2.5 fraction is 92.7%.

The emission estimates were not based on direct measurements of pollutant concentrations during the fire and, consequently, were not converted to normal conditions. The obtained estimates were subsequently used as input data for a dispersion model, and predicted atmospheric concentrations were calculated for the meteorological conditions recorded during the actual fire.

### 2.2. Modelling the Dispersion Range of Pollutants

Once the estimated quantities of gases and particulates emitted into the atmosphere during waste incineration had been determined, their likely concentrations were calculated at the nearest locations where people regularly spend time and who might be exposed to the harmful effects of these substances. For this purpose, the Gaussian plume model and Pasquill atmospheric stability classes were used. This model constitutes the reference method recommended by applicable Polish regulations. Guidelines for calculating pollutant dispersion using the Gaussian plume model and Pasquill atmospheric stability classes are set out in Annex 3 to the Regulation of the Minister of the Environment of 26 January 2010 on reference values for certain substances in the air (Journal of Laws of 2010, No. 16, item 87). [10]. However, the use of this specific calculation approach is not mandatory across the entire European Union under a single legal act. EU legislation sets out common requirements regarding the assessment of ambient air quality and the use of modelling methods, while leaving Member States free to choose the specific modelling methods and tools used to meet those requirements. In many countries, the use of models based on Pasquill atmospheric stability classes stems from national regulations or official regulatory guidelines. In Germany, the relevant legal instrument—the Technical Instructions on Air Quality Control (*Technische Anleitung zur Reinhaltung der Luft*—TA Luft), issued under the Federal Immission Control Act (BImSchG)—mandates the use of the AUSTAL2000 model as the reference method for calculating pollutant dispersion [23,24]. Although the document itself no longer explicitly names the Pasquill model, AUSTAL2000 is derived from classical dispersion models based on Pasquill’s theory. This method is explicitly cited in the regulations of the US Environmental Protection Agency (EPA) set forth in 40 CFR Part 51 [25]. Conversely, in other countries and their respective guidelines—such as those developed by the UK Environment Agency—the Pasquill atmospheric stability class is used exclusively [26]. The model employed assumes meteorological conditions from the measurement period, homogeneous terrain, constant emission parameters, and a simplified description of atmospheric turbulence [27]. Despite its simplicity, it continues to be used in preliminary analyses and reference methodologies.

### 2.3. Assessment of Environmental Risk

Polish regulations govern the concentrations of hazardous substances released into the environment by establishing reference levels. Exceeding these levels results in a deterioration of air quality. To assess the risk posed to humans and the environment by air pollution involving toxic substances, it is necessary to evaluate the concentrations of these substances in the context of environmental damage and adverse effects on human health. Pollution levels indicating a deterioration in air quality may pose a potential threat to the environment only once quality standards are exceeded. However, exceeding these values does not necessarily equate to an immediate threat to human health. Regarding ambient air quality in Poland, the Regulation of the Minister of the Environment of 24 August 2012 on levels of certain substances in the air (Journal of Laws 2012, item 1031, as amended) establishes limit values for specific substances in the air—expressed as concentrations—as well as the permissible frequency of exceedances for hourly concentrations within a year [28].

To assess the risk to humans, it is necessary to consider the concentrations of toxic compounds whose short-term exposure can be harmful to the body [29]. These values have been partially established in the Regulation of the Minister of Family, Labour and Social Policy of 12 June 2018 on the maximum permissible concentrations and intensities of agents harmful to health in the work environment (Journal of Laws 2018, item 1286, as amended) [30], based on defined maximum permissible concentrations (NDS)—understood as the time-weighted average concentration to which an employee is exposed during an 8-hour daily and average weekly working period over the course of their entire employment, and which should not cause adverse effects on their health or the health of future generations.

Under Polish regulations, a deterioration in air quality may be deemed significant if the frequency of exceeding maximum reference values or permissible concentration levels for substances in the air exceeds 0.274% of the time annually for sulfur dioxide and 0.2% of the time annually for other substances. This condition applies to the concentrations of regulated substances. The 0.2% annual time threshold corresponds to 17.5 h, whereas the waste fire in the case under analysis lasted a maximum of 3.5 h. Consequently, any exceedances of the limit values used to define air quality deterioration during this period fell within the permissible limit of 0.2% of the time annually. This means that the regulatory condition in question does not apply to the case at hand.

Given that applicable regulations do not specify limit values for all substances emitted during the fire, an analysis of limit values was also conducted based on standards established by the EPA (U.S. Environmental Protection Agency)—the air quality standard applicable in the United States—and OSHA (Occupational Safety and Health Administration)—the occupational safety and health standard applicable in the United States. Establishing additional, reliable reference values is justified when assessing the risk associated with the potential impact of toxic air emissions on human health. Based on the data analysis, a summary of information regarding the pollutants under consideration was prepared (Table 2). 

Consequently, exceeding the values considered permissible according to the aforementioned sources of information should be regarded as a justified environmental risk entailing a threat to human health. However, it should be noted that the most reliable confirmation of such risk is obtained by referencing the NDS value, as well as the RfC and STEL values, which are relevant to the potential health risks faced by individuals exposed to specific air pollutant concentrations over a short-term period. Since the case study in question concerns a short-term event, comparing the emissions in question against annual exposure levels is not appropriate.

## 3. Results

**Figure 1:**
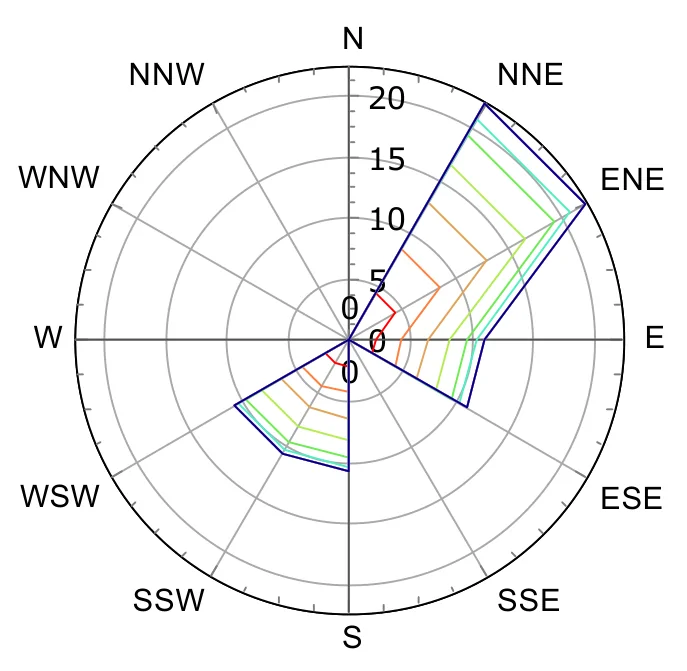
The wind rose adopted for the calculations.

Given that this analysis focuses on the emission episode (the duration of the fire), data regarding the wind rose (wind directions and speeds) during the analyzed emission period were also taken into account. Meteorological data indicate a predominance of winds blowing from the north-northeast. The wind rose used in the calculations is presented below (Figure 1). The aerodynamic roughness coefficient was calculated in accordance with regulations [10] as the weighted average of the surface roughness of the various terrain types surrounding the analyzed site, assuming a value of z0 = 0.035.

Emission source parameters were determined based on data regarding the waste fire’s surface area, which was assumed to be 50 m^2^. As part of the modeling calculations, additional concentration monitoring points were established in the immediate vicinity (i.e., an additional grid of points), extending to inhabited areas such as the nearest identified settlement. These points were established to determine concentration levels in areas occupied by people and, consequently, to assess the degree of public exposure to pollutants. The nearest sensitive areas—specifically the aforementioned settlement—are located approximately 700 m from the fire site. Due to the temporary nature of the emission (a fire lasting 3.5 h), annual average concentrations are not taken into account.

Concentration isolines are presented below, alongside tabular data specifying the maximum value and the frequency of exceeding the “D1” parameter—which represents the reference value or permissible level of a given substance in the air (averaged over one hour). The height of the calculation plane (“Z”), receptor coordinates, the resulting pollutant concentrations at those points, and the meteorological conditions critical to the obtained concentration values. This latter parameter was determined based on established atmospheric stability classes corresponding to the wind speed range defined as the “critical wind speed” (Table 3).

Calculations presented in Figure 2, Figure 3, Figure 4, Figure 5, Figure 6, Figure 7, Figure 8, Figure 9, Figure 10, Figure 11, Figure 12, Figure 13, Figure 14, Figure 15, Figure 16, Figure 17, Figure 18, Figure 19 and Figure 20 indicated that the concentrations of substances emitted as a result of the waste fire in question, recorded in the nearest residential area including the nearest designated locality:

- do not exceed applicable national air quality standards specified in the Regulation of the Minister of the Environment of 26 January 2010 on reference values for certain substances in the air (Journal of Laws 2010, No. 16, item 87) [10];
- do not exceed air quality standards established by the EPA;
- do not exceed air quality standards established by OSHA (Occupational Safety and Health Administration);
- do not exceed the NDS values (highest permissible concentrations) specified in the Regulation of the Minister of Family, Labour and Social Policy of 12 June 2018 on the highest permissible concentrations and intensities of factors harmful to health in the work environment (Journal of Laws 2018, item 1286, as amended) [30].

Modeling analysis presented in Table 4, Table 5, Table 6, Table 7, Table 8, Table 9, Table 10, Table 11, Table 12, Table 13, Table 14, Table 15, Table 16, Table 17, Table 18, Table 19, Table 20, Table 21 and Table 22 determined that the atmospheric conditions most unfavorable for the dispersion of pollutants near sensitive areas were an atmospheric stability class of "6—stable," a critical wind speed of 1 m/s, and a critical wind direction of "S—south." This means that the highest atmospheric pollutant concentrations during the fire were recorded under these meteorological conditions. Nevertheless, calculations indicate that even under these adverse meteorological conditions, pollution levels did not reach values that could affect the health or lives of residents in the nearest settlement. This conclusion pertains to the short-term exposure of individuals located 700 meters from the incident site. The values and findings in question cannot be applied to pollution concentrations occurring in the immediate vicinity of the waste fire. The analysis focused on the exposure risk for local residents resulting from the operations and waste storage activities; consequently, the occupational risk to firefighters conducting the firefighting operation was not assessed.

**Figure 2:**
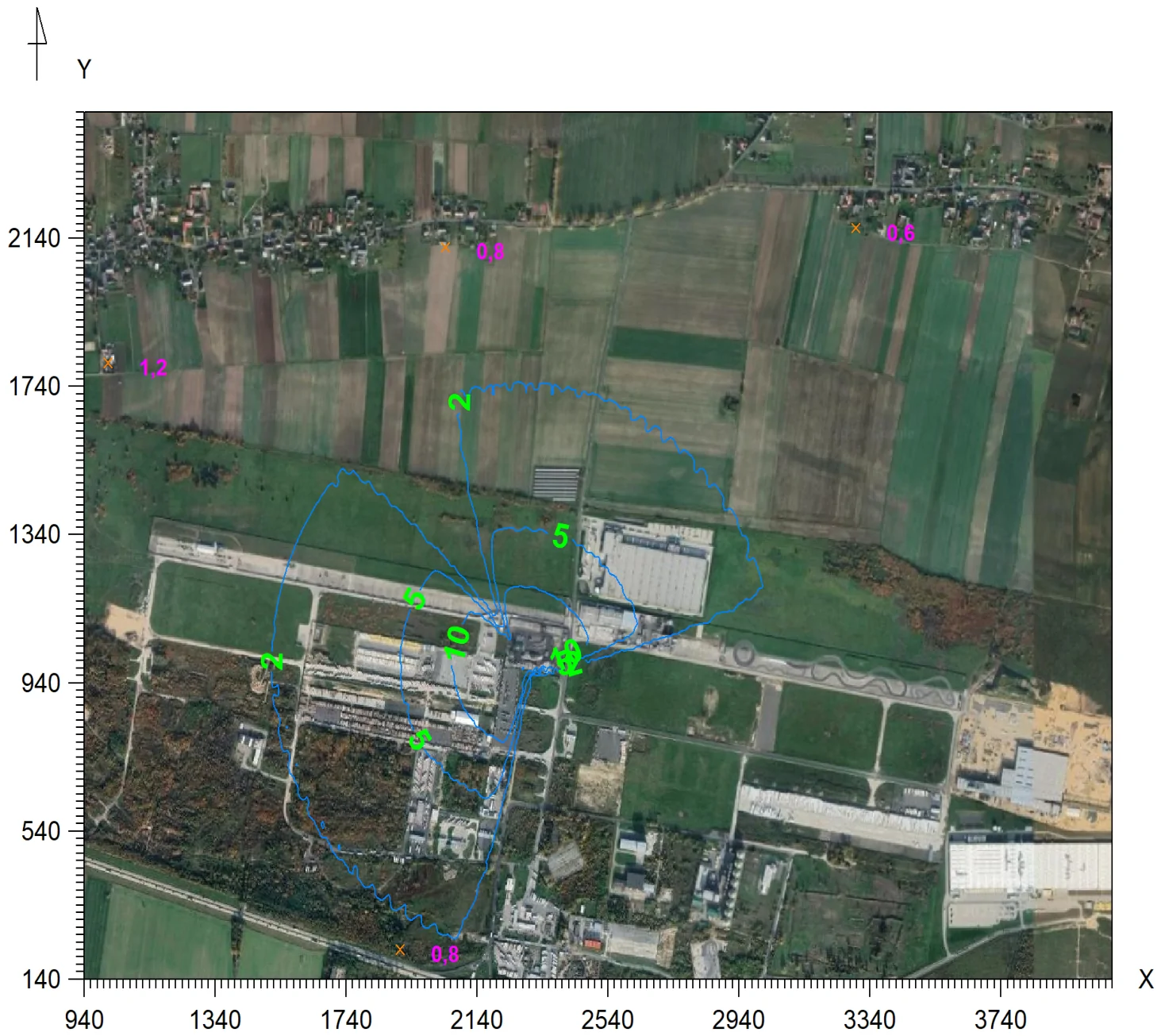
Isolines of maximum concentrations particulate matter PM-10. Chart key:  0,0X—Pollutant concentration values [µg/m^3^],  ----—Isolines of maximum concentrations [µg/m^3^], 0,00X—Maximum concentration values at calculation points (receptor grid) [µg/m^3^].

**Table 4:** Summary of maximum particulate matter PM-10 concentration (µg/m^3^) values in the supplementary grid.

Parameter	Value	X	Y	Z	crit.	crit.	crit.
		m	m	m	stab.clas	wind.s.	wind.d.
Maximum concentration µg/m^3^	1.2000	1012	1804	1	6	1	S
Frequency of exceedances D1 = 280, %	0.0000	-	-	-	-	-	-

**Figure 3:**
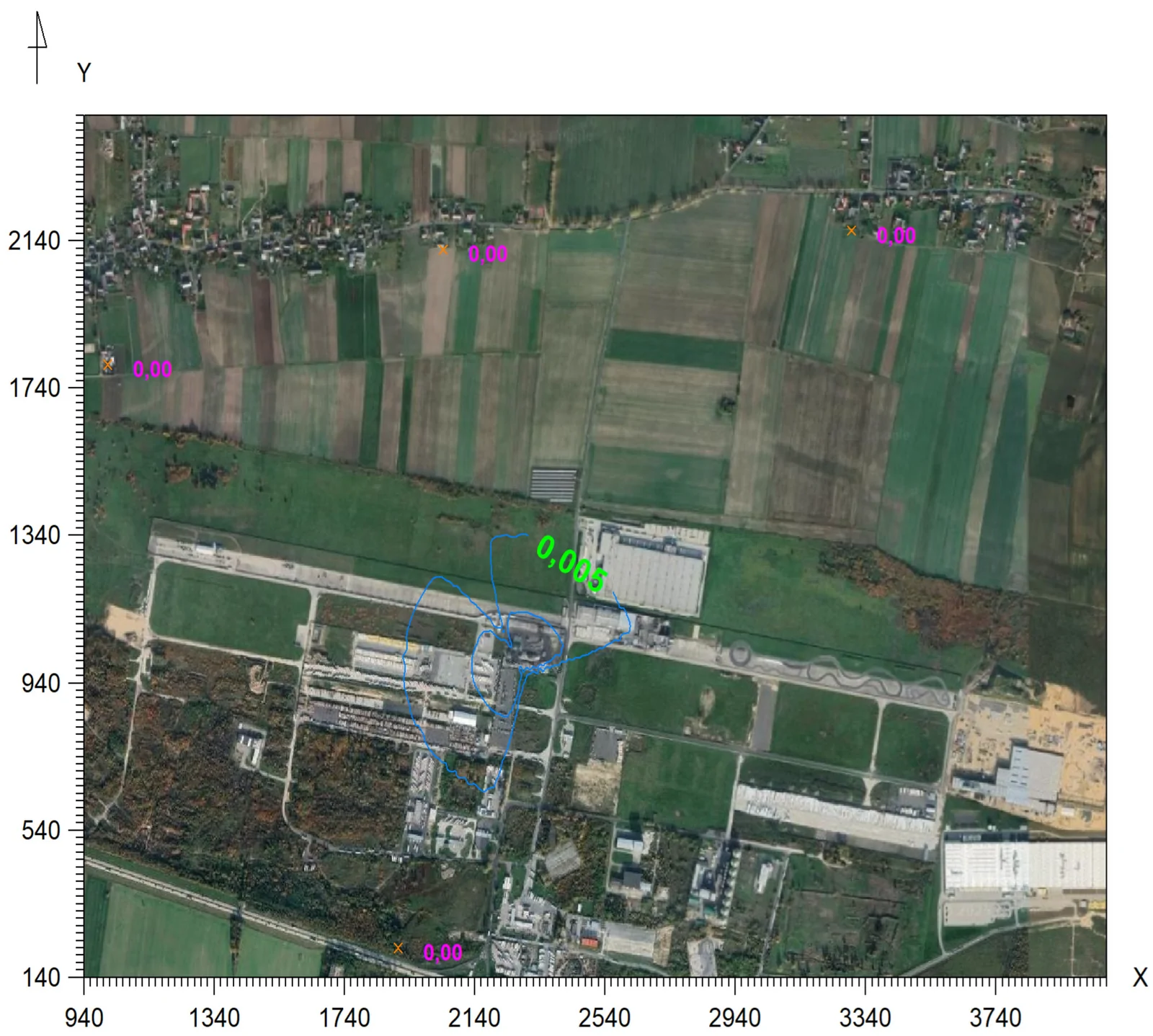
Isolines of maximum concentrations Benzo[e]pyrene. Chart key:  0,0X—Pollutant concentration values [µg/m^3^],  ----—Isolines of maximum concentrations [µg/m^3^], 0,00X—Maximum concentration values at calculation points (receptor grid) [µg/m^3^].

**Table 5:** Summary of maximum Benzo[e]pyrene concentrations (µg/m^3^) in the supplementary grid.

Parameter	Value	X	Y	Z	crit.	crit.	crit.
		m	m	m	stab.clas	wind.s.	wind.d.
Maximum concentration µg/m^3^	0.0000	1012	1804	1	6	1	S
Frequency of exceedances D1 = 0.012, %	0.0000	-	-	-	-	-	-

**Figure 4:**
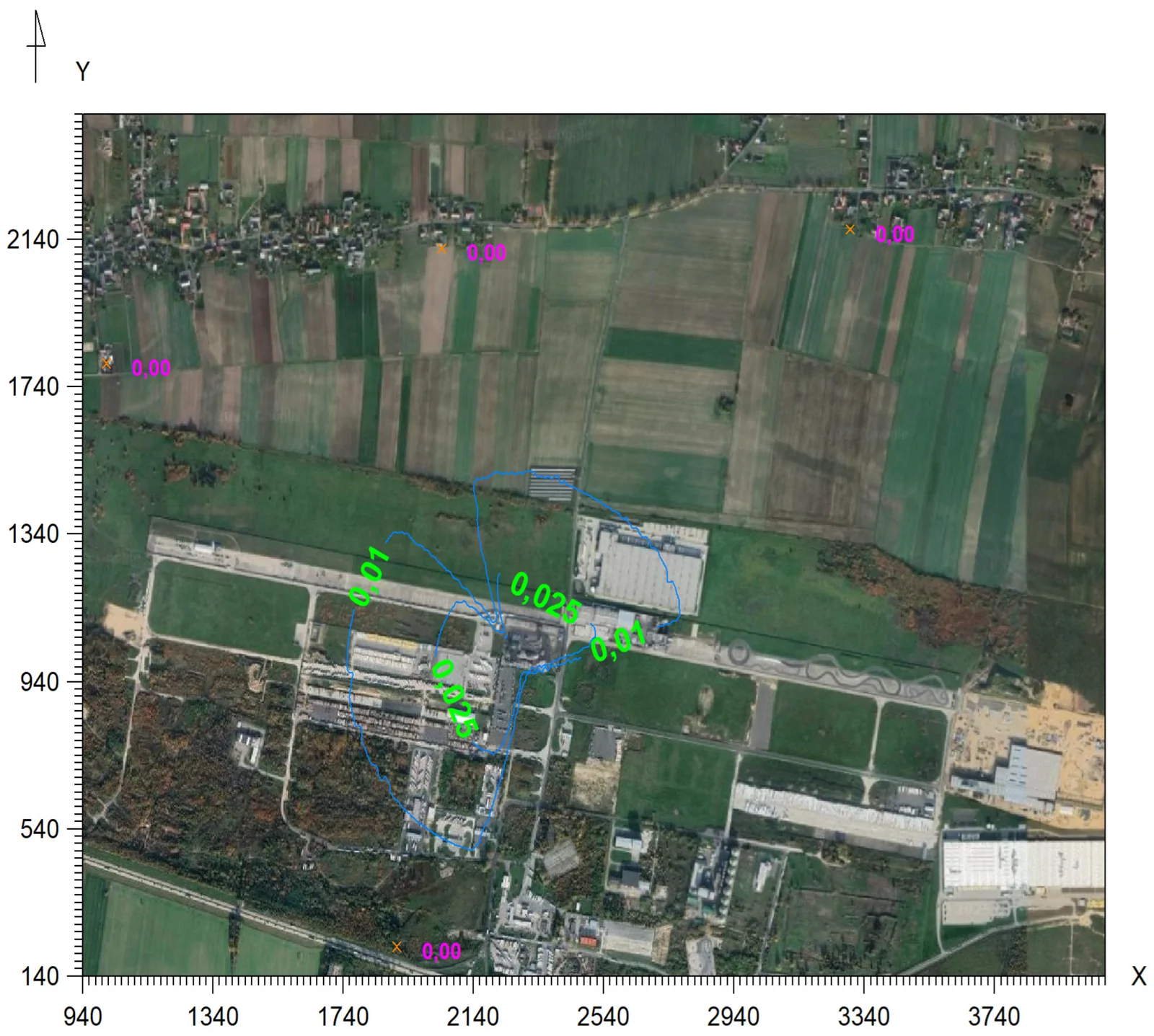
Isolines of maximum concentrations Benzene. Chart key:  0,0X—Pollutant concentration values [µg/m^3^],  ----—Isolines of maximum concentrations [µg/m^3^], 0,00X—Maximum concentration values at calculation points (receptor grid) [µg/m^3^].

**Table 6:** Summary of maximum benzene concentration (µg/m^3^) values in the supplementary grid.

Parameter	Value	X	Y	Z	crit.	crit.	crit.
		m	m	m	stab.clas	wind.s.	wind.d.
Maximum concentration µg/m^3^	0.0000	1012	1804	1	6	1	S
Frequency of exceedances D1 = 30, %	0.0000	-	-	-	-	-	-

**Figure 5:**
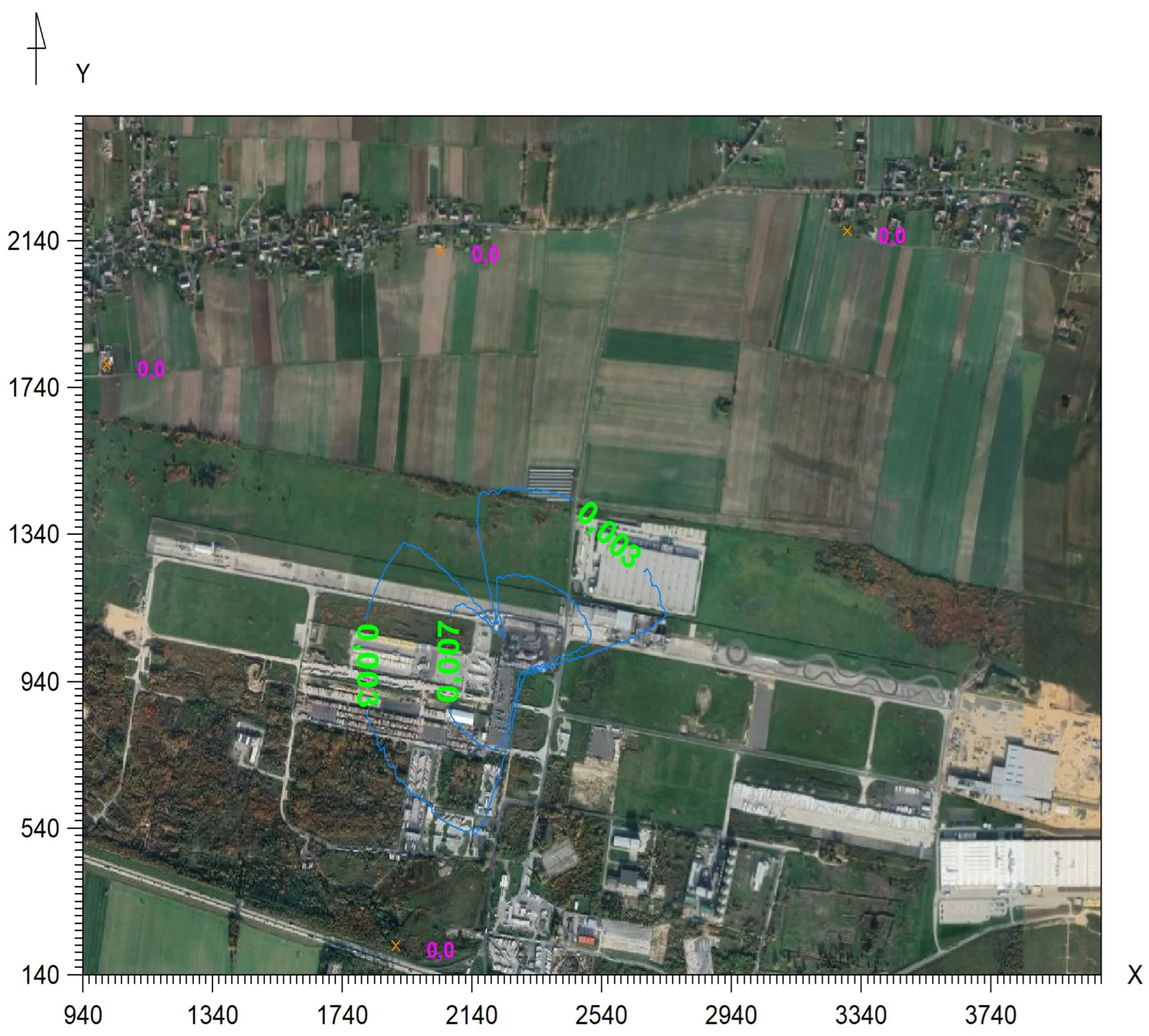
Isolines of maximum concentrations Toluene. Chart key:  0,0X—Pollutant concentration values [µg/m^3^],  ----—Isolines of maximum concentrations [µg/m^3^], 0,00X—Maximum concentration values at calculation points (receptor grid) [µg/m^3^].

**Table 7:** Summary of maximum toluene concentration (µg/m^3^) values in the supplementary grid.

Parameter	Value	X	Y	Z	crit.	crit.	crit.
		m	m	m	stab.clas	wind.s.	wind.d.
Maximum concentration µg/m^3^	0.0000	1012	1804	1	6	1	S
Frequency of exceedances D1 = 100, %	0.0000	-	-	-	-	-	-

**Figure 6:**
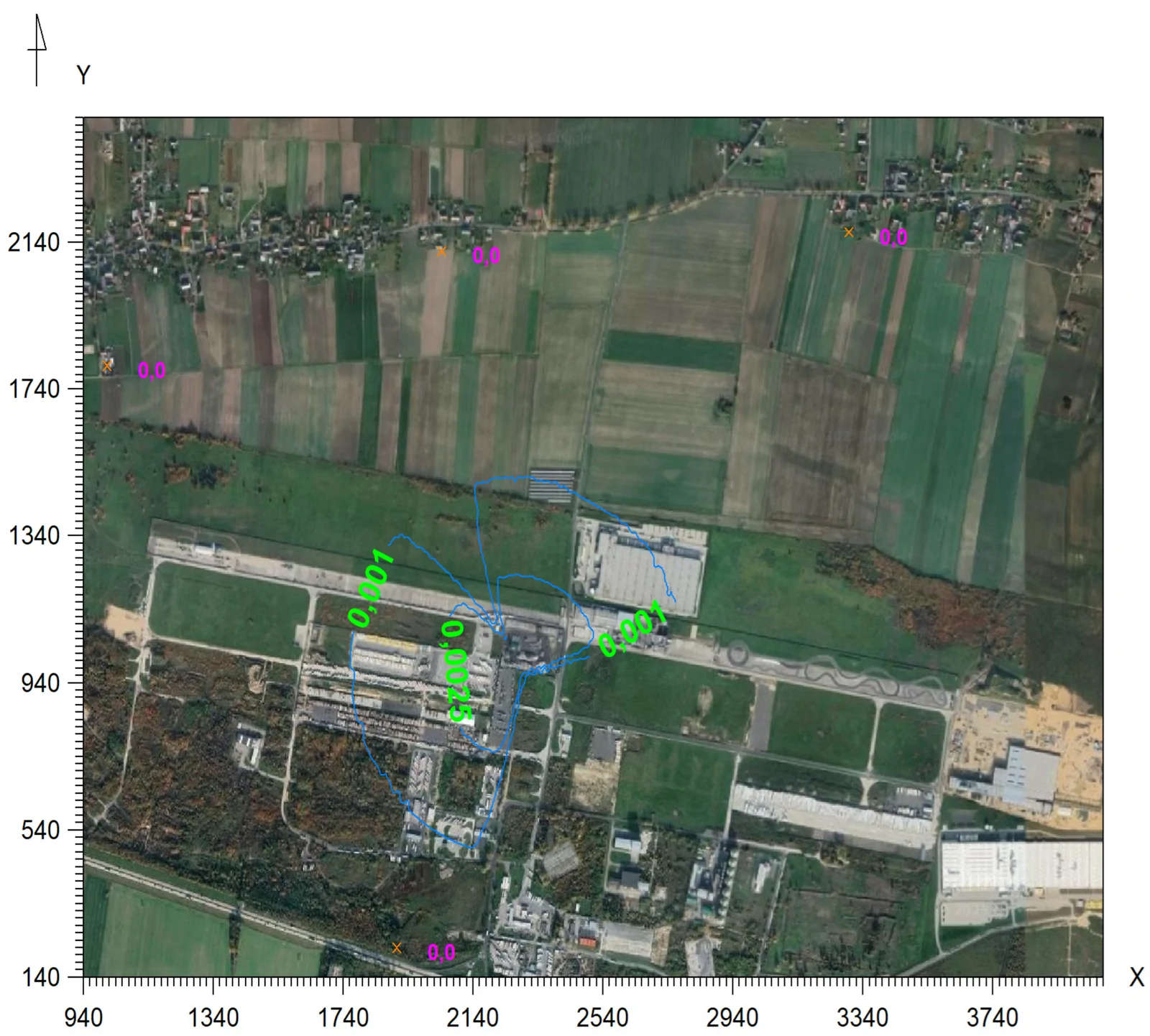
Isolines of maximum concentrations Ethylbenzene. Chart key:  0,0X—Pollutant concentration values [µg/m^3^],  ----—Isolines of maximum concentrations [µg/m^3^], 0,00X—Maximum concentration values at calculation points (receptor grid) [µg/m^3^].

**Table 8:** Summary of maximum ethylbenzene concentrations (µg/m^3^) in the supplementary grid.

Parameter	Value	X	Y	Z	crit.	crit.	crit.
		m	m	m	stab.clas	wind.s.	wind.d.
Maximum concentration µg/m^3^	0.0000	1012	1804	1	6	1	S
Frequency of exceedances D1 = 500, %	0.0000	-	-	-	-	-	-

**Figure 7:**
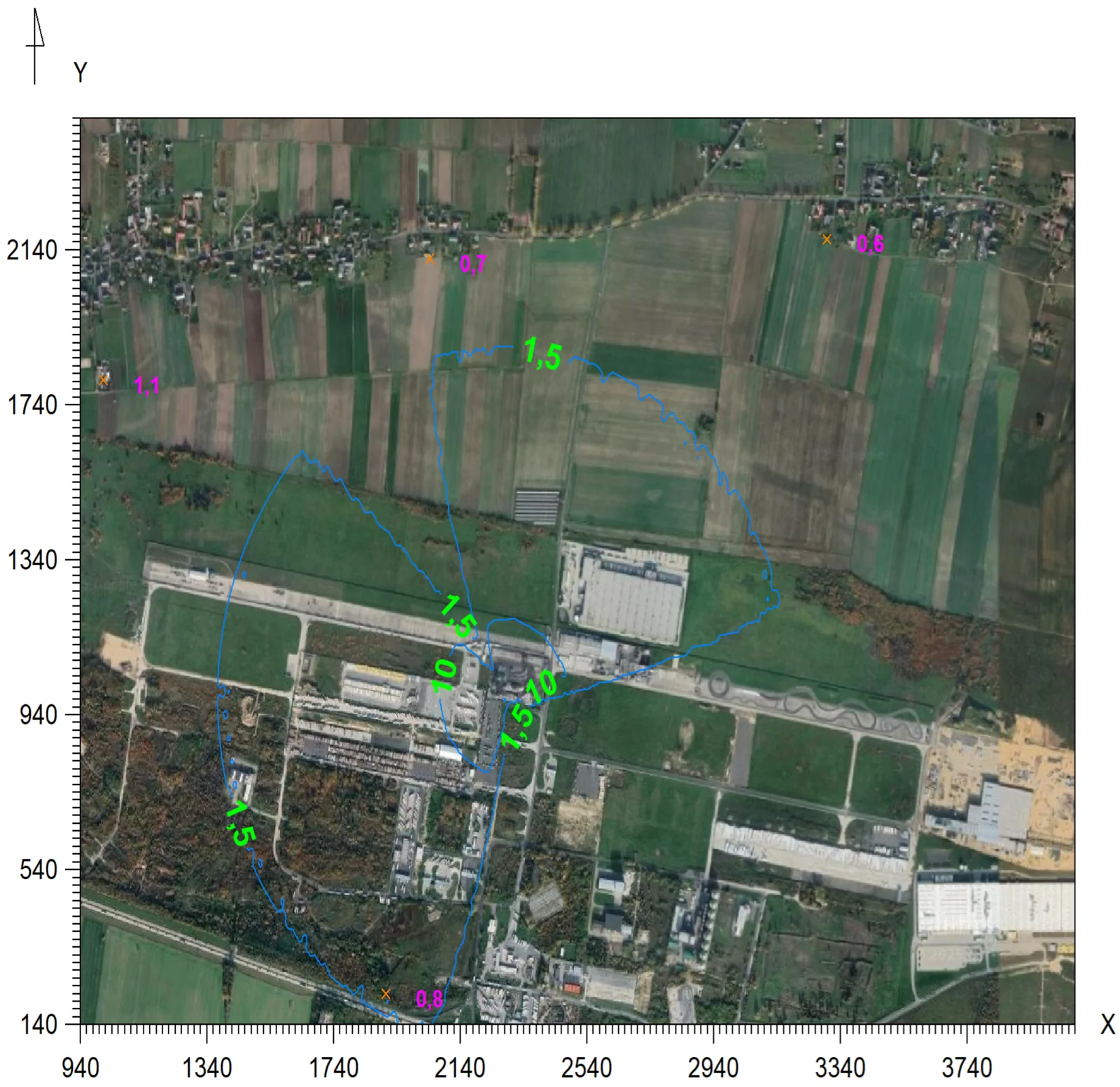
Isolines of maximum concentrations PM 2.5 particulate matter. Chart key:  0,0X—Pollutant concentration values [µg/m^3^],  ----—Isolines of maximum concentrations [µg/m^3^], 0,00X—Maximum concentration values at calculation points (receptor grid) [µg/m^3^].

**Table 9:** Summary of maximum PM 2.5 concentration (µg/m^3^) values in the supplementary grid.

Parameter	Value	X	Y	Z	crit.	crit.	crit.
		m	m	m	stab.clas	wind.s.	wind.d.
Maximum concentration µg/m^3^	1.1000	1012	1804	1	6	1	S
Frequency of exceedances—not applicable, D1 none	-	-	-	-	-	-	-

**Figure 8:**
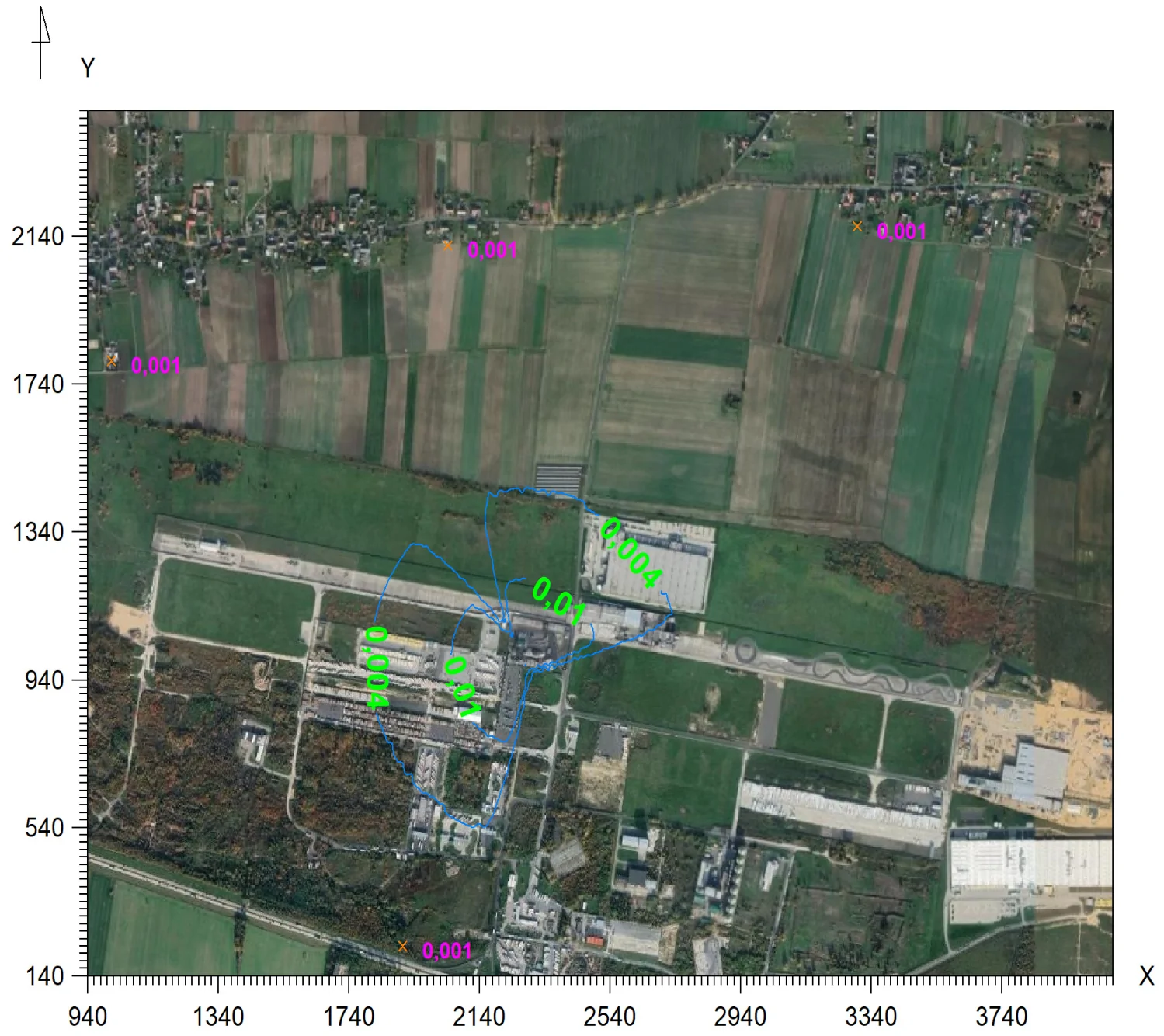
Isolines of maximum concentrations Hexane. Chart key:  0,0X—Pollutant concentration values [µg/m^3^],  ----—Isolines of maximum concentrations [µg/m^3^], 0,00X—Maximum concentration values at calculation points (receptor grid) [µg/m^3^].

**Table 10:** Summary of maximum hexane concentration (µg/m^3^) values in the supplementary grid.

Parameter	Value	X	Y	Z	crit.	crit.	crit.
		m	m	m	stab.clas	wind.s.	wind.d.
Maximum concentration µg/m^3^	0.0010	1012	1804	1	6	1	S
Frequency of exceedances D1 = 700, %	0.0000	-	-	-	-	-	-

**Figure 9:**
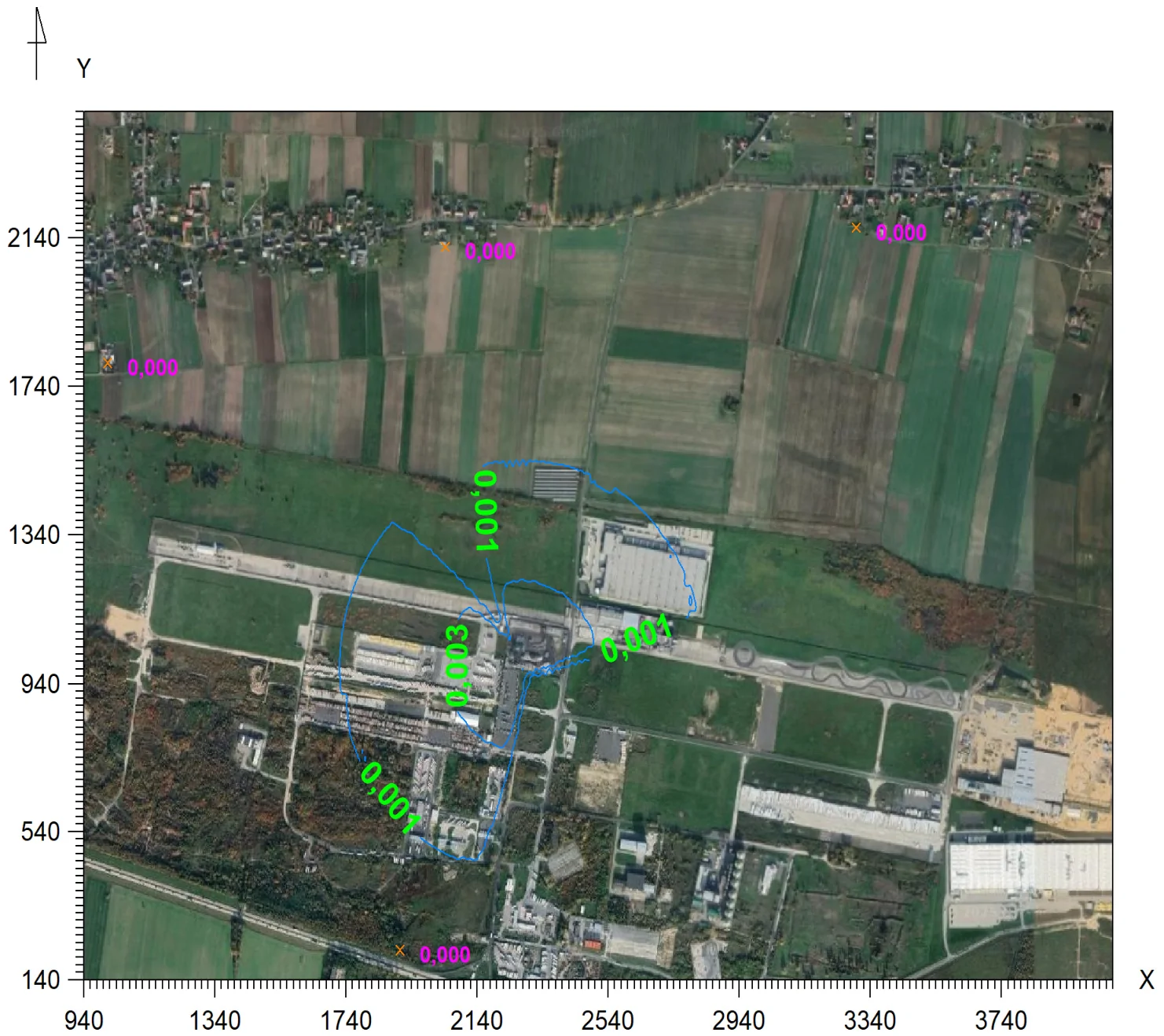
Isolines of maximum concentrations Anthracene. Chart key:  0,0X—Pollutant concentration values [µg/m^3^],  ----—Isolines of maximum concentrations [µg/m^3^], 0,00X—Maximum concentration values at calculation points (receptor grid) [µg/m^3^].

**Table 11:** Summary of maximum anthracene concentration (µg/m^3^) values in the supplementary grid.

Parameter	Value	X	Y	Z	crit.	crit.	crit.
		m	m	m	stab.clas	wind.s.	wind.d.
Maximum concentration µg/m^3^	0.0010	1012	1804	1	6	1	S
Frequency of exceedances D1 = 200, %	0.0000	-	-	-	-	-	-

**Figure 10:**
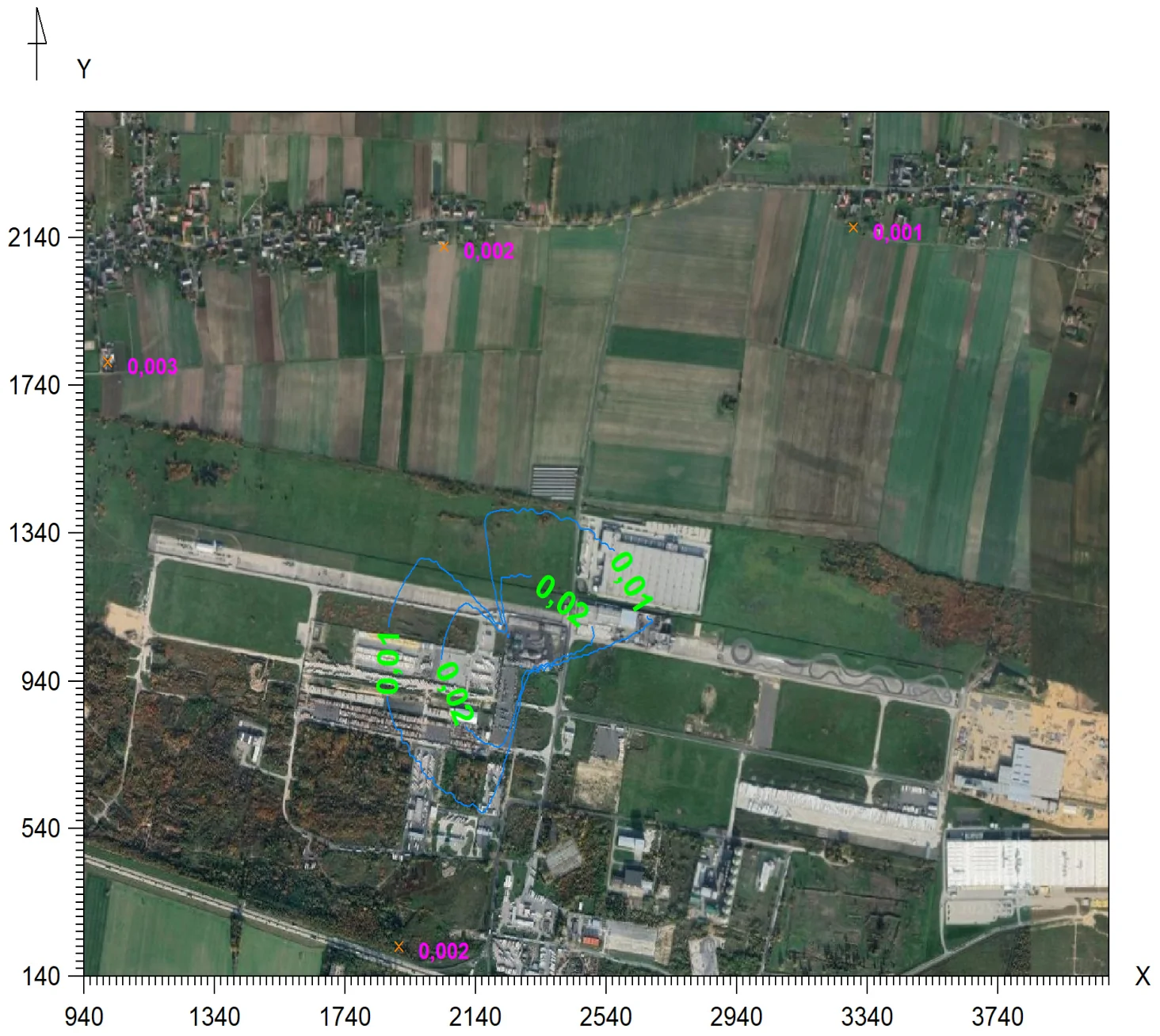
Isolines of maximum concentrations Benzo[b]fluoranthene. Chart key:  0,0X—Pollutant concentration values [µg/m^3^],  ----—Isolines of maximum concentrations [µg/m^3^], 0,00X—Maximum concentration values at calculation points (receptor grid) [µg/m^3^].

**Table 12:** Summary of maximum Benzo[b]fluoranthene concentrations (µg/m^3^) in the supplementary grid.

Parameter	Value	X	Y	Z	crit.	crit.	crit.
		m	m	m	stab.clas	wind.s.	wind.d.
Maximum concentration µg/m^3^	0.0030	1012	1804	1	6	1	S
Frequency of exceedances D1 = 20, %	0.0000	-	-	-	-	-	-

**Figure 11:**
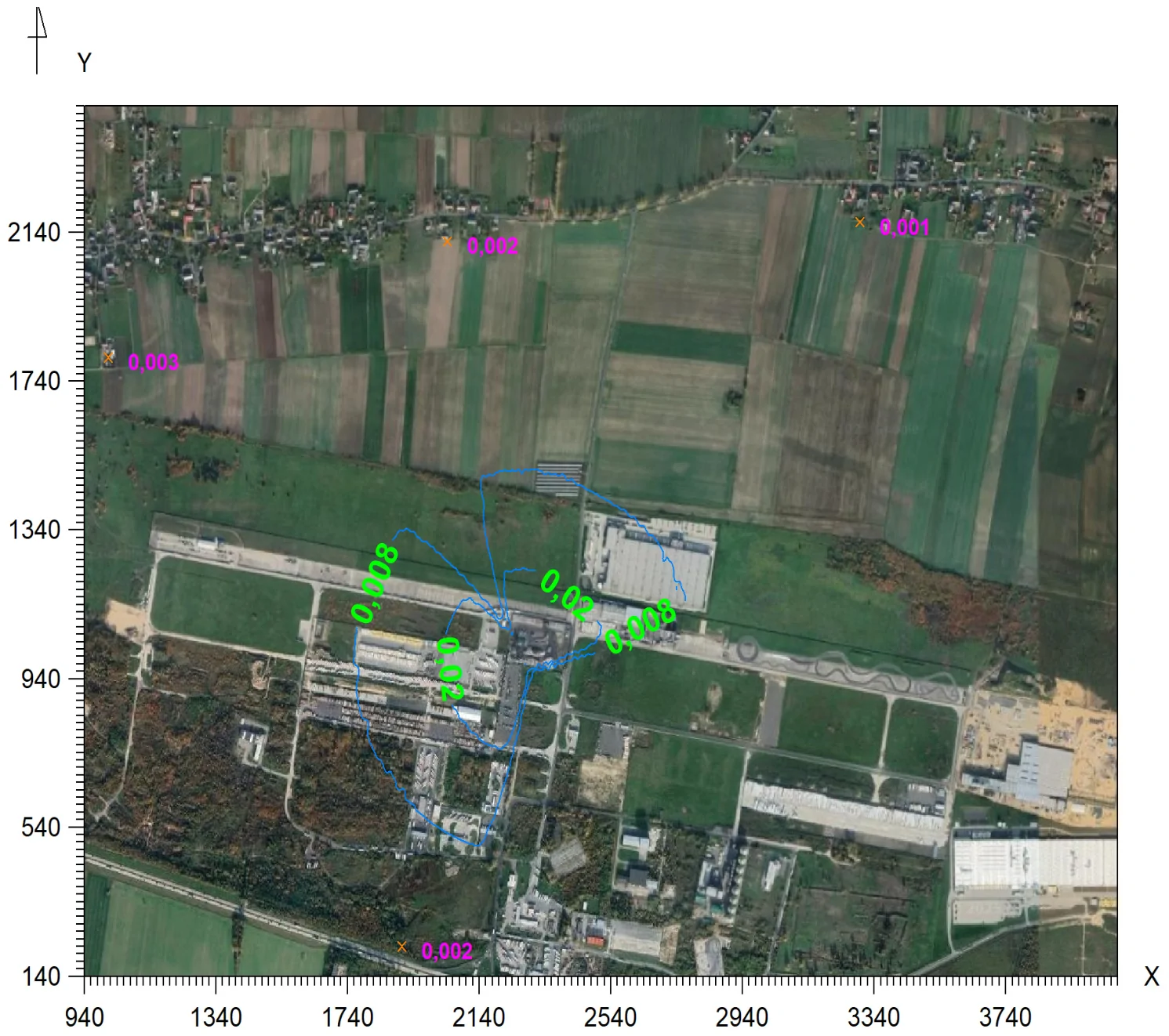
Isolines of maximum concentrations Benzo[e]pyrene. Chart key:  0,0X—Pollutant concentration values [µg/m^3^],  ----—Isolines of maximum concentrations [µg/m^3^], 0,00X—Maximum concentration values at calculation points (receptor grid) [µg/m^3^].

**Table 13:** Summary of maximum Benzo[e]pyrene concentration (µg/m^3^) values in the supplementary grid.

Parameter	Value	X	Y	Z	crit.	crit.	crit.
		m	m	m	stab.clas	wind.s.	wind.d.
Maximum concentration µg/m^3^	0.0030	1012	1804	1	6	1	S
Frequency of exceedances D1 = 0.008, %	0.0000	-	-	-	-	-	-

**Figure 12:**
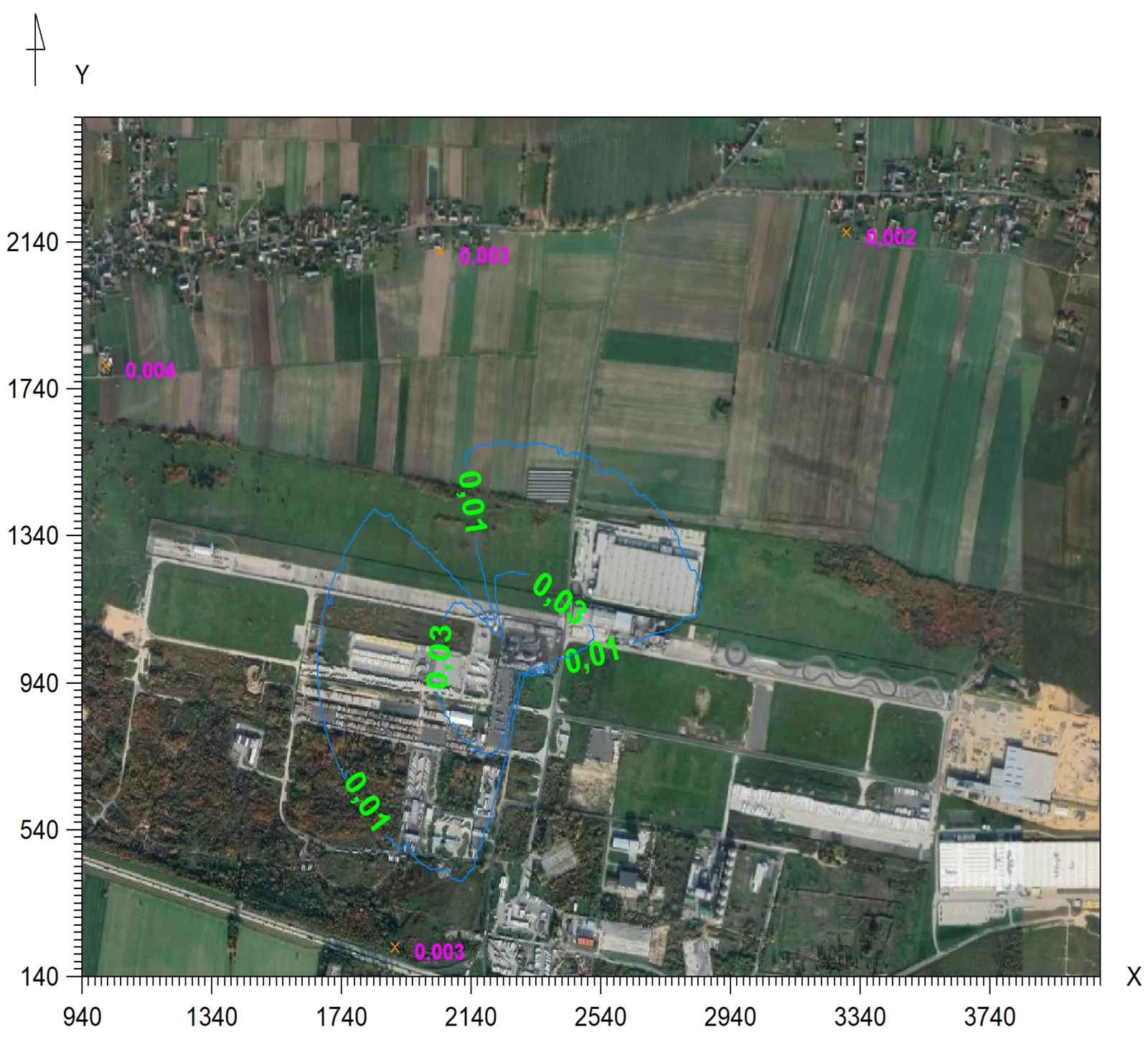
Isolines of maximum concentrations Benzo[g,h,i]perylene. Chart key:  0,0X—Pollutant concentration values [µg/m^3^],  ----—Isolines of maximum concentrations [µg/m^3^], 0,00X—Maximum concentration values at calculation points (receptor grid) [µg/m^3^].

**Table 14:** Summary of maximum Benzo[g,h,i]perylene concentrations (µg/m^3^) in the supplementary grid.

Parameter	Value	X	Y	Z	crit.	crit.	crit.
		m	m	m	stab.clas	wind.s.	wind.d.
Maximum concentration µg/m^3^	0.0040	1012	1804	1	6	1	S
Frequency of exceedances D1 = 200, %	0.0000	-	-	-	-	-	-

**Figure 13:**
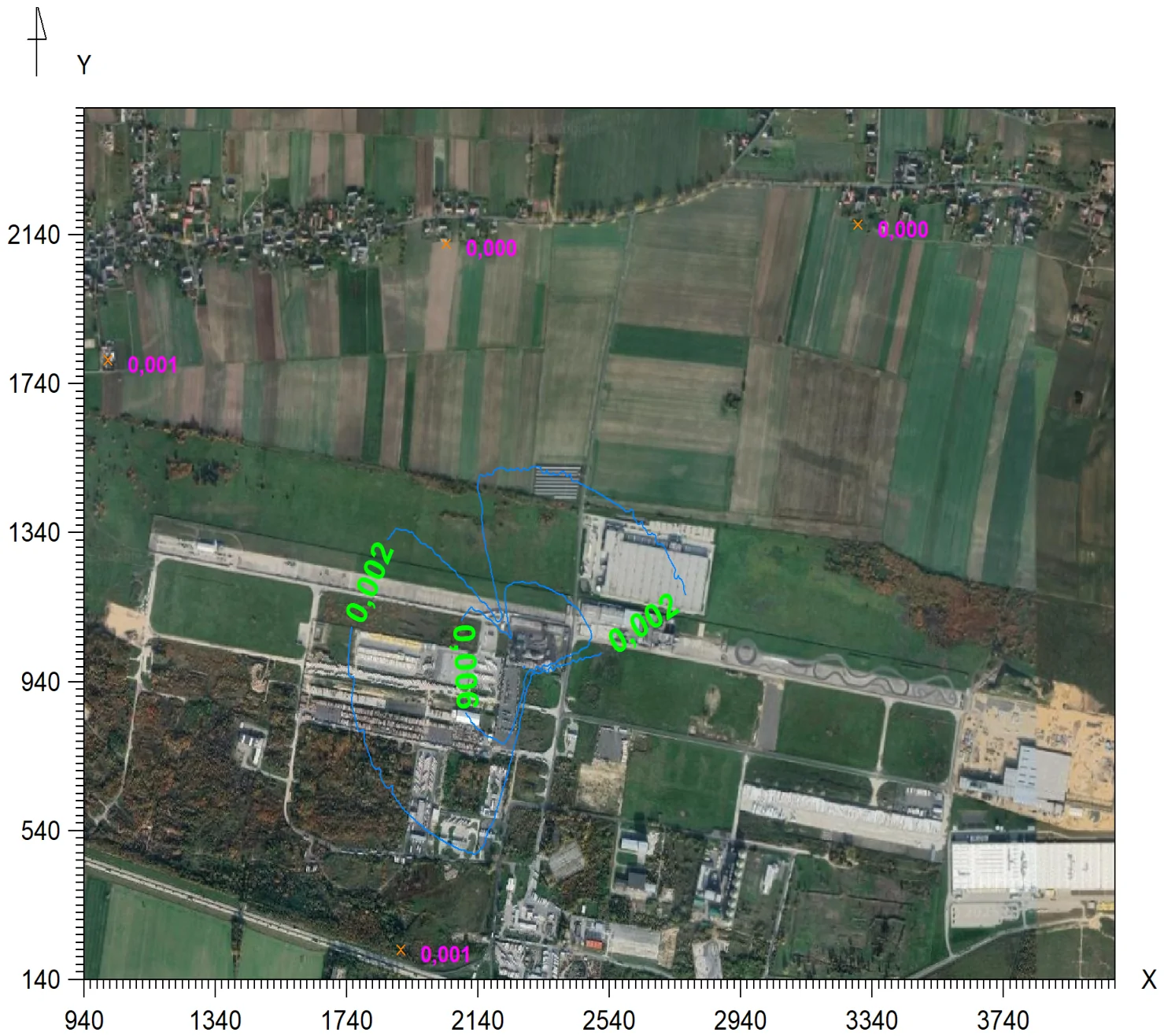
Isolines of maximum concentrations Benzo[k]fluoranthene. Chart key:  0,0X—Pollutant concentration values [µg/m^3^],  ----—Isolines of maximum concentrations [µg/m^3^], 0,00X—Maximum concentration values at calculation points (receptor grid) [µg/m^3^].

**Table 15:** Summary of maximum Benzo[k]fluoranthene concentrations (µg/m^3^) in the supplementary grid.

Parameter	Value	X	Y	Z	crit.	crit.	crit.
		m	m	m	stab.clas	wind.s.	wind.d.
Maximum concentration µg/m^3^	0.0010	1012	1804	1	6	1	S
Frequency of exceedances D1 = 200, %	0.0000	-	-	-	-	-	-

**Figure 14:**
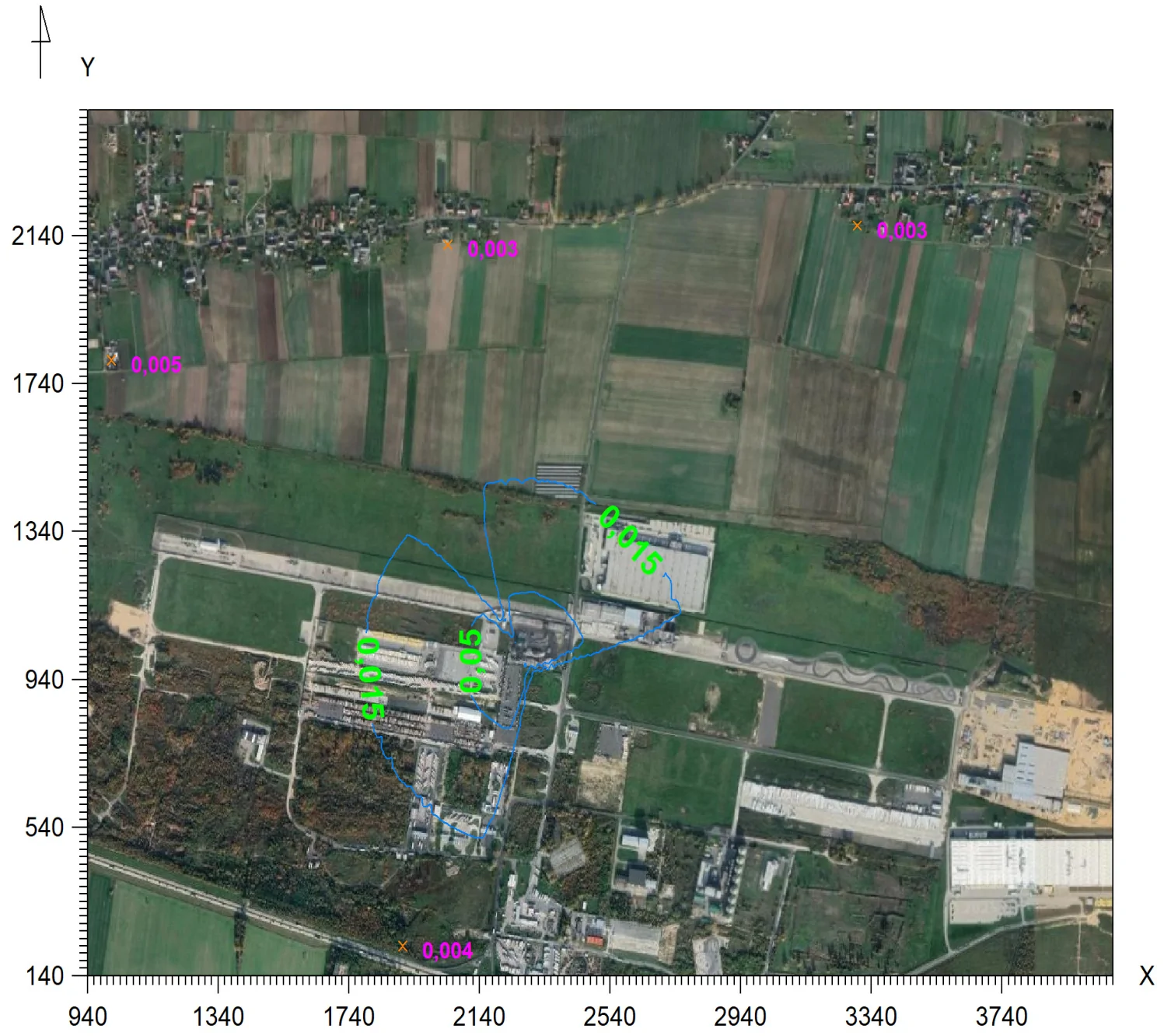
Isolines of maximum concentrations Chrysene. Chart key:  0,0X—Pollutant concentration values [µg/m^3^],  ----—Isolines of maximum concentrations [µg/m^3^], 0,00X—Maximum concentration values at calculation points (receptor grid) [µg/m^3^].

**Table 16:** Summary of maximum chrysen concentrations (µg/m^3^) in the supplementary grid.

Parameter	Value	X	Y	Z	crit.	crit.	crit.
		m	m	m	stab.clas	wind.s.	wind.d.
Maximum concentration µg/m^3^	0.0050	1012	1804	1	6	1	S
Frequency of exceedances D1 = 20, %	0.0000	-	-	-	-	-	-

**Figure 15:**
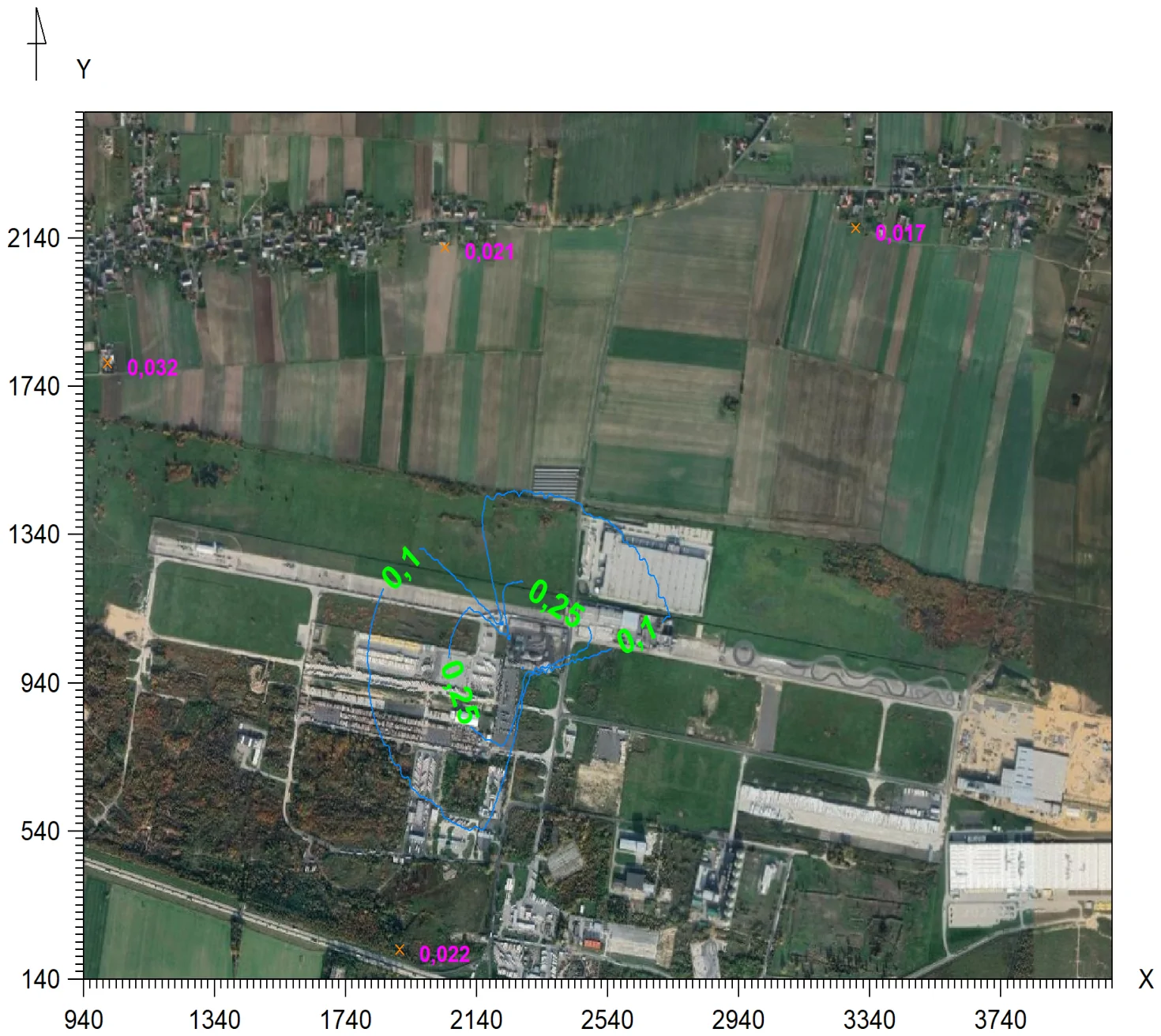
Isolines of maximum concentrations Fluoranthene. Chart key:  0,0X—Pollutant concentration values [µg/m^3^],  ----—Isolines of maximum concentrations [µg/m^3^], 0,00X—Maximum concentration values at calculation points (receptor grid) [µg/m^3^].

**Table 17:** Summary of maximum fluoranthene concentrations (µg/m^3^) in the supplementary grid.

Parameter	Value	X	Y	Z	crit.	crit.	crit.
		m	m	m	stab.clas	wind.s.	wind.d.
Maximum concentration µg/m^3^	0.0320	1012	1804	1	6	1	S
Frequency of exceedances D1 = 200, %	0.0000	-	-	-	-	-	-

**Figure 16:**
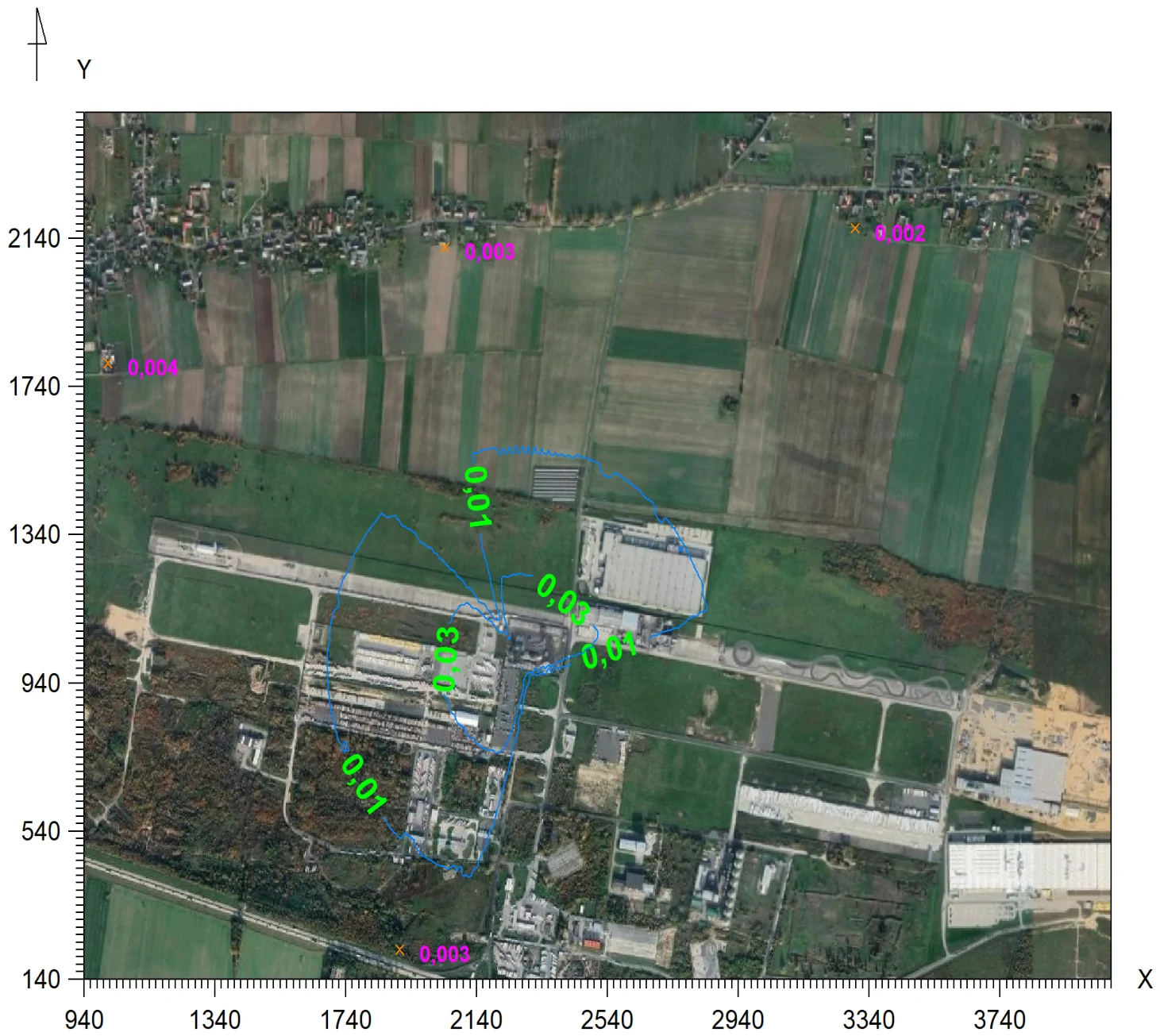
Isolines of maximum concentrations Benz[a]anthracene. Chart key:  0,0X—Pollutant concentration values [µg/m^3^],  ----—Isolines of maximum concentrations [µg/m^3^], 0,00X—Maximum concentration values at calculation points (receptor grid) [µg/m^3^].

**Table 18:** Summary of maximum Benz[a]anthracene concentrations (µg/m^3^) in the supplementary grid.

Parameter	Value	X	Y	Z	crit.	crit.	crit.
		m	m	m	stab.clas	wind.s.	wind.d.
Maximum concentration µg/m^3^	0.0020	1012	1804	1	6	1	S
Frequency of exceedances D1 = 0.2, %	0.0000	-	-	-	-	-	-

**Figure 17:**
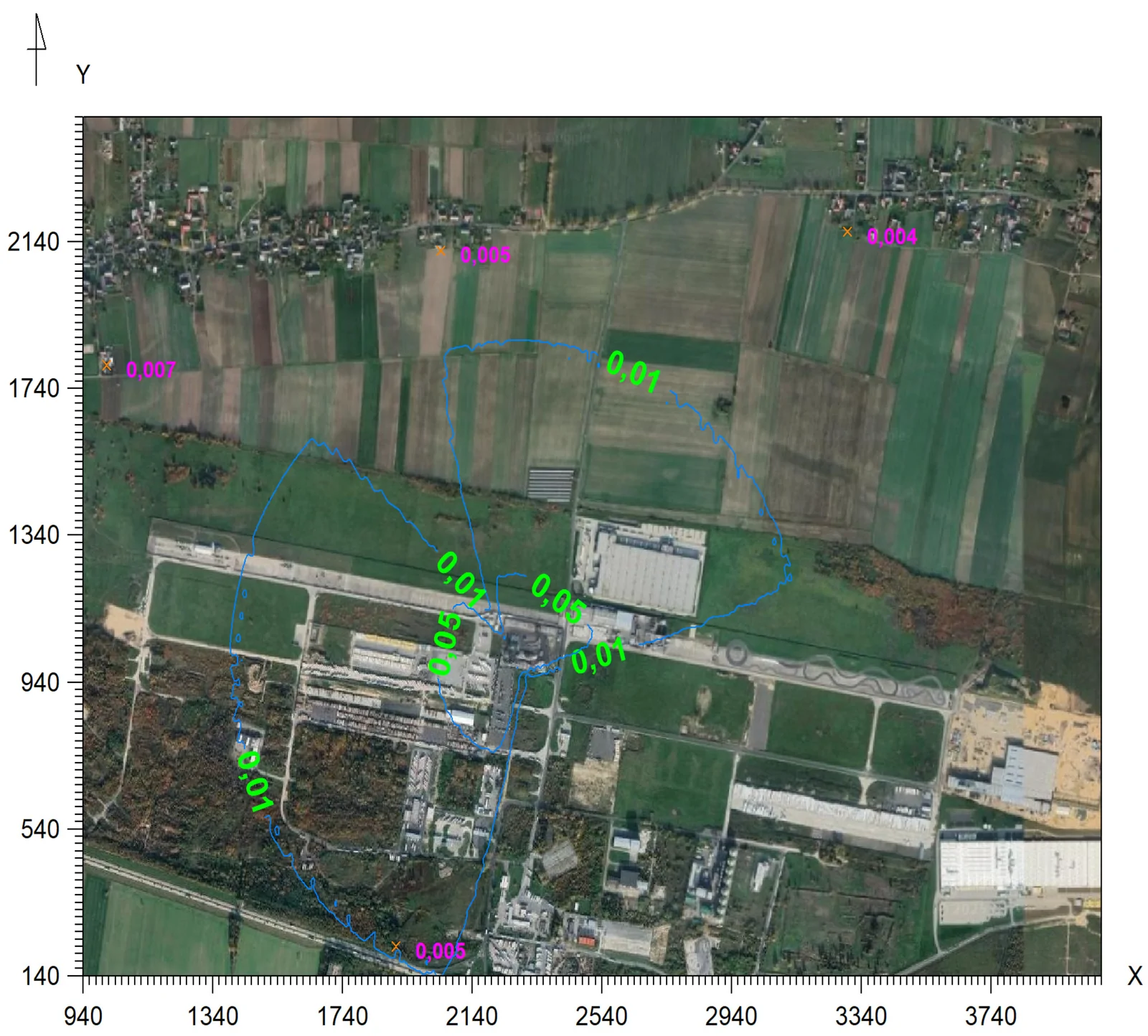
Isolines of maximum concentrations Phenanthrene. Chart key:  0,0X—Pollutant concentration values [µg/m^3^],  ----—Isolines of maximum concentrations [µg/m^3^], 0,00X—Maximum concentration values at calculation points (receptor grid) [µg/m^3^].

**Table 19:** Summary of maximum phenanthrene concentrations (µg/m^3^) in the supplementary grid.

Parameter	Value	X	Y	Z	crit.	crit.	crit.
		m	m	m	stab.clas	wind.s.	wind.d.
Maximum concentration µg/m^3^	0.0070	1012	1804	1	6	1	S
Frequency of exceedances D1 = 200, %	0.0000	-	-	-	-	-	-

**Figure 18:**
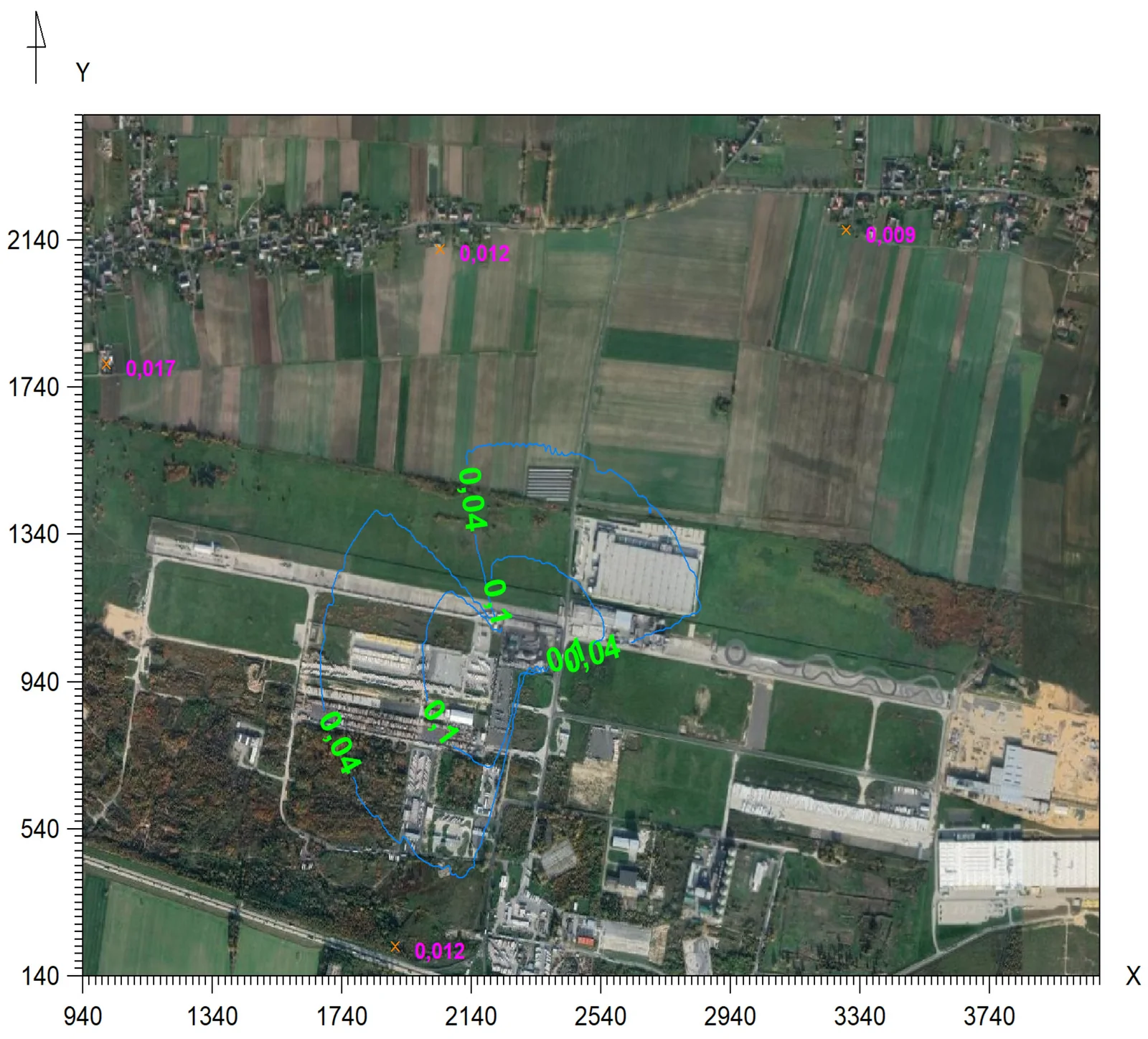
Isolines of maximum concentrations Pyrene. Chart key:  0,0X—Pollutant concentration values [µg/m^3^],  ----—Isolines of maximum concentrations [µg/m^3^], 0,00X—Maximum concentration values at calculation points (receptor grid) [µg/m^3^].

**Table 20:** Summary of maximum pyrene concentrations (µg/m^3^) in the supplementary grid.

Parameter	Value	X	Y	Z	crit.	crit.	crit.
		m	m	m	stab.clas	wind.s.	wind.d.
Maximum concentration µg/m^3^	0.0170	1012	1804	1	6	1	S
Frequency of exceedances D1 = 200, %	0.0000	-	-	-	-	-	-

**Figure 19:**
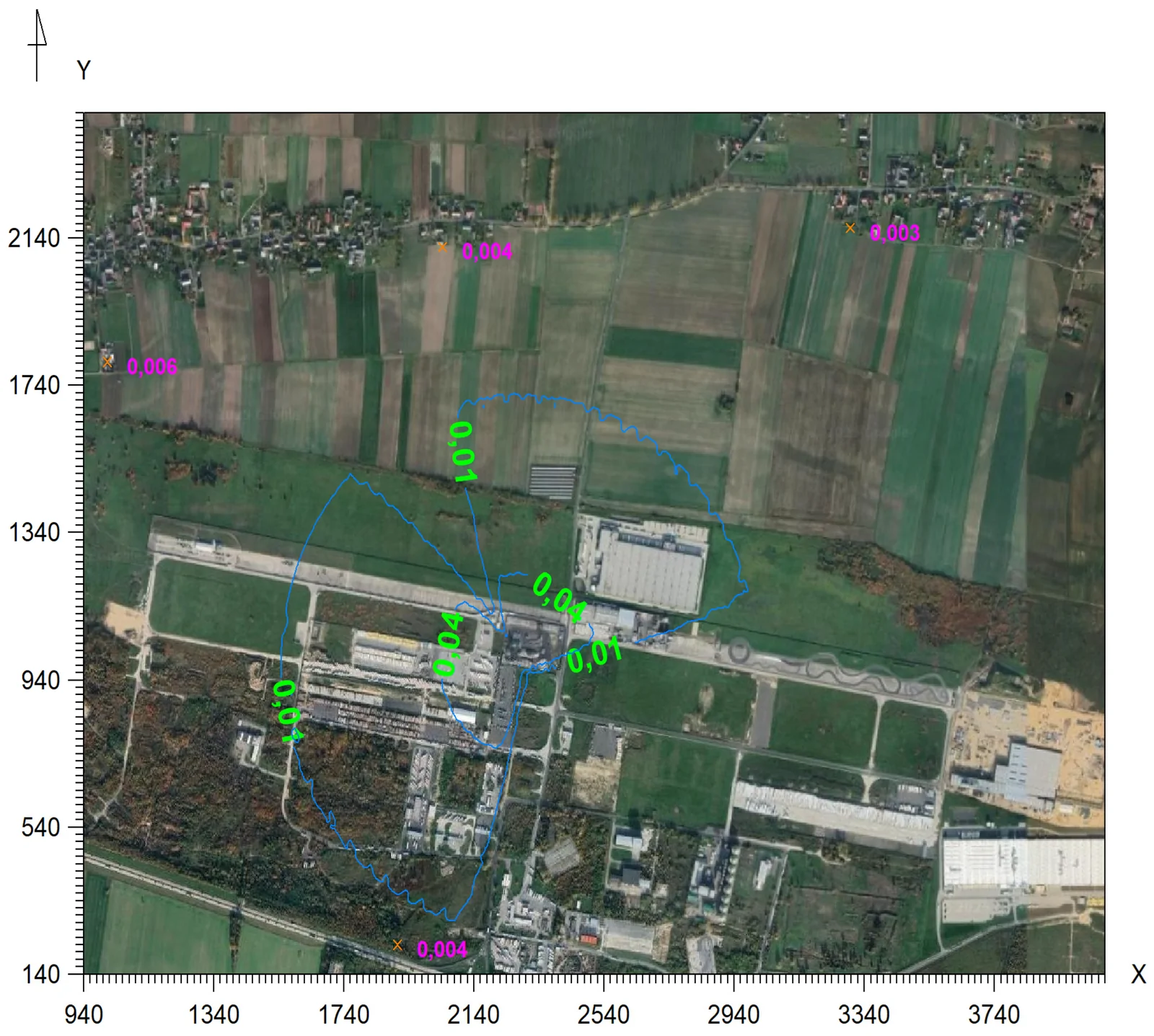
Isolines of maximum concentrations Retene. Chart key:  0,0X—Pollutant concentration values [µg/m^3^],  ----—Isolines of maximum concentrations [µg/m^3^], 0,00X—Maximum concentration values at calculation points (receptor grid) [µg/m^3^].

**Table 21:** Summary of maximum retene concentrations (µg/m^3^) in the supplementary grid.

Parameter	Value	X	Y	Z	crit.	crit.	crit.
		m	m	m	stab.clas	wind.s.	wind.d.
Maximum concentration µg/m^3^	0.0060	1012	1804	1	6	1	S
Frequency of exceedances—not applicable, D1 none	-	-	-	-	-	-	-

**Figure 20:**
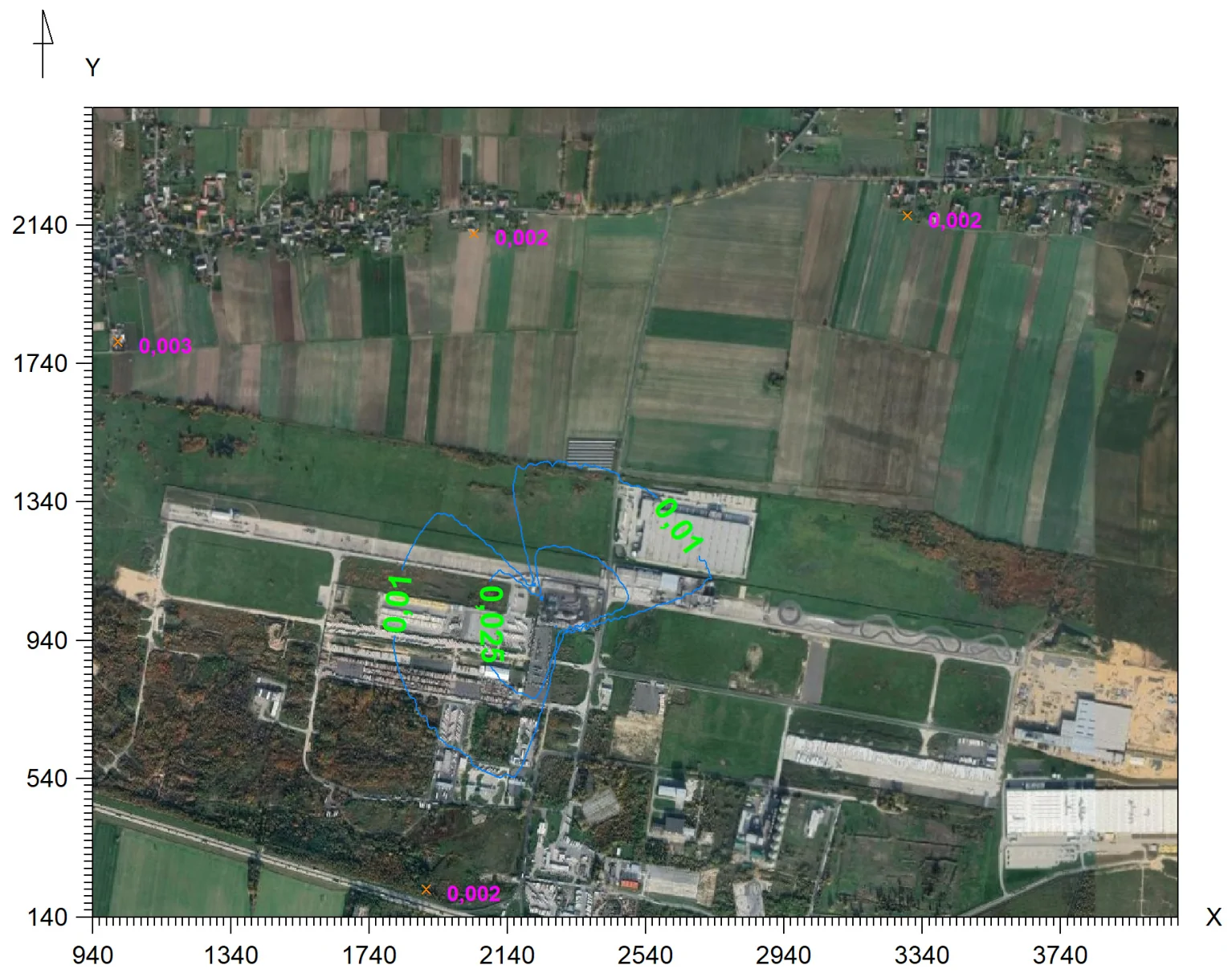
Isolines of maximum concentrations Indeno[1,2,3-cd]pyrene. Chart key:  0,0X—Pollutant concentration values [µg/m^3^],  ----—Isolines of maximum concentrations [µg/m^3^], 0,00X—Maximum concentration values at calculation points (receptor grid) [µg/m^3^].

**Table 22:** Summary of maximum Indeno[1,2,3-cd]pyrene concentrations (µg/m^3^) in the supplementary grid.

Parameter	Value	X	Y	Z	crit.	crit.	crit.
		m	m	m	stab.clas	wind.s.	wind.d.
Maximum concentration µg/m^3^	0.0030	1012	1804	1	6	1	S
Frequency of exceedances D1 = 0.2, %	0.0000	1012	1804	1	6	1	S

## 4. Discussion

Based on the analyses and calculations performed, no evidence was found to indicate that the waste accumulated at the site of the uncontrolled burning was stored in quantities or under conditions posing a threat to human life or health, as it was not demonstrated that:

- in areas surrounding the nearest population centers, concentrations of substances generated during the uncontrolled burning of waste could reach levels toxic to humans upon exposure,
- persons were present in areas outside the nearest population centers—and particularly in the immediate vicinity of the burning waste—who could have been exposed to harmful substances produced by the fire.

The analysis results also indicate that a fire of this scale could not have caused a deterioration in air quality, as there was no evidence that concentrations of hazardous substances emitted into the atmosphere during the fire exceeded the reference values specified in the Regulation of the Minister of the Environment of 26 January 2010 on reference values for certain substances in the air (Journal of Laws 2010 No. 16, item 87) [10].

Determining the actual health impact of uncontrolled waste-burning incidents is difficult for a number of reasons. Such incidents occur unexpectedly, making it impossible to conduct comprehensive measurements of the concentrations of pollutants generated by the waste combustion and emitted into the environment. Under such conditions, neither combustion temperatures nor process dynamics are measured, as the event is uncontrolled. Based on information regarding the incident, it is possible to determine the duration of the fire, its location, and the time the firefighting operation concluded. Documentation and data concerning the actual accumulation of waste allow for an estimation of the approximate quantity of waste involved. This, in turn, enables an estimate of the approximate amount of waste burned. Using the information gathered in this way, it is necessary to determine whether the incident posed a threat to human life or health. The answer to this question should depend on the quantity of pollutants released into the environment due to the uncontrolled burning of waste, the extent of their dispersion, and the degree of human exposure to specific concentrations of toxic substances.

It cannot be assumed that every instance of exceeding a limit value constitutes a threat to human life or health, as everything depends on the quantity of the substance absorbed. However, prolonged exposure to toxic substances can be harmful to health. For this reason, the release of even a minimal amount of a harmful substance into the environment cannot be definitively considered a threat to human life or health.

In the case under analysis, no exceedance of health-relevant concentrations of toxic substances in the ambient air was found—resulting from short-term exposure—at a distance of 700 meters from the residential buildings located closest to the fire site. The high number of waste fires and the associated environmental risks—stemming from excessive emissions of toxic substances in recent years—highlight an urgent need for experimental work to identify the actual products of waste combustion occurring at low temperatures and with limited oxygen availability. Such studies should focus primarily on mixed municipal waste, the combustible fraction of municipal waste, and batteries and accumulators. Analyzing environmental risk regarding the toxicity of air emissions is also crucial for the legal aspects of pursuing claims related to violations of environmental quality standards, including risks to human health.

## 5. Conclusions

This study presents a method for assessing human exposure to pollutants released during an uncontrolled waste fire, based on a case study involving pollutant dispersion modeling and a comparison of predicted concentrations against relevant reference values (toxicological and regulatory). The assessment accounts for key factors influencing exposure, such as the type and quantity of waste burned, the duration of the fire, and meteorological conditions.

In the analyzed case—involving the uncontrolled burning of 14,000 kg of municipal waste processing residues over a period of 3.5 h—predicted concentrations at monitoring points located in a residential area (approximately 700 m from the fire site) did not exceed applicable Polish and international reference values for the meteorological scenario analyzed. These results indicate that, under the assumed conditions, no significant risk of exposure to the pollutants under study was identified at the assessed residential locations. However, these results should not be interpreted as ruling out potential health or environmental risks in the immediate vicinity of the fire or under different emission and meteorological conditions.

A significant limitation of the assessment is the uncertainty associated with the emission inventory, stemming from the uncontrolled and unpredictable nature of the fire and the lack of precise information regarding the composition of the burned waste. Consequently, the predicted concentrations should be treated as scenario-based estimates rather than a direct reflection of actual exposure. The proposed method, focused on analysis at points of exposure (receptors), demonstrates the utility of pollutant dispersion modeling for the rapid preliminary assessment of environmental and human health risks associated with uncontrolled waste fires, particularly in situations where direct measurements of emissions or air quality are unavailable. Its application can facilitate the identification of areas of potential exposure and serve as a basis for a subsequent, more detailed risk assessment. 

## Figures and Tables

**Table 1:** Calculations of emissions resulting from a fire.

Substances	Emission Factor, [mg/kg]	Quantity of Waste Incinerated, [kg]	Total Emissions, [kg]	Duration of the Fire, [h]	Hourly Emission, [kg/h]
Benzene	0.01230	14,000	0.00017220	3.5	0.0000492
Toluene	0.00330		0.00004620		0.0000132
Ethylbenzene	0.00120		0.00001680		0.0000048
Hexane	0.00430		0.00006020		0.0000172
Anthracene	0.00132		0.00001848		0.0000053
Benzo[a]pyrene	0.00753		0.00010542		0.0000301
Benzo[b]fluoranthene	0.00925		0.00012950		0.0000370
Benzo[e]pyrene	0.00965		0.00013510		0.0000386
Benzo[g,h,i]perylene	0.01493		0.00020902		0.0000597
Benzo[k]fluoranthene	0.00251		0.00003514		0.0000100
Benz[a]anthracene	0.01441		0.00020174		0.0000576
Chrysene	0.01718		0.00024052		0.0000687
Fluoranthene	0.10705		0.00149870		0.0004282
Indeno[1,2,3-cd]pyrene	0.01070		0.00014980		0.0000428
Phenanthrene	0.02405		0.00033670		0.0000962
Pyrene	0.05881		0.00082334		0.0002352
Retene	0.01877		0.00026278		0.0000751
Total particulate matter	8.00000		0.11200000		0.0320000

**Table 2:** Comparison of permissible concentrations, reference values, and standards for the substances included in the analysis.

Substance	Reference Value as Set Out in the Regulation Journal of Laws 2010 [10], No. 16, Item 87 [µg/m^3^]	NDS in Accordance with the Regulation Journal of Laws 2018, Item 1286 [µg/m^3^] [30]	Standard According to EPA [µg/m^3^]	Standard According to OSHA [µg/m^3^]
Benzene	30	1600	30 (IRIS RfC)	320 (REL-STEL)
Toluene	100	100,000	5000 (IRIS RfC)	no data
Ethylbenzene	500	200,000	1000 (IRIS RfC)	435,000 (PEL-TWA)
Hexane	no data	72,000	700 (IRIS RfC)	180,000 (PEL-TWA)
Anthracene	no data	no data	no data	200 (PEL-TWA)
Benzo[a]pyrene	0.012	2	0.002 (IRIS RfC)	200 (PEL-TWA)
Benzo[b]fluoranthene	no data	no data	20 (IRIS RfC)	200 (PEL-TWA)
Benzo[e]pyrene	no data	no data	0.008 (IRIS RfC)	200 (PEL-TWA)
Benzo[g,h,i]perylene	no data	no data	no data	200 (PEL-TWA)
Benzo[k]fluoranthene	no data	no data	no data	200 (PEL-TWA)
Benz[a]anthracene	no data	no data	0.2 (IRIS RfC)	200 (PEL-TWA)
Chrysene	no data	no data	20 (IRIS RfC)	200 (PEL-TWA)
Fluoranthene	no data	no data	no data	200 (PEL-TWA)
Indeno[1,2,3-cd]pyrene	no data	no data	0.2 (IRIS RfC)	200 (PEL-TWA)
Phenanthrene	no data	no data	no data	200 (PEL-TWA)
Pyrene	no data	no data	no data	200 (PEL-TWA)
Retene	no data	no data	no data	no data
Total particulate matter	280	1460	no data	no data

**Table 3:** Established atmospheric stability classes in relation to wind speed ranges.

Stability Classes	Critical Wind Speed Range u_a_ x [m/s]
1—extremely unstable	1–3
2—unstable	1–5
3—slightly unstable	1–8
4—neutral	1–11
5—slightly stable	1–5
6—stable	1–4

## Data Availability

The original contributions presented in this study are included in the article. Further inquiries can be directed to the corresponding author.

## Abbreviations

The following abbreviations are used in this manuscript:

NIK	Najwyższa Izba Kontroli (The Supreme Audit Office)
EPA	Environmental Protection Agency
NDS	Najwyższe dopuszczalne stężenie (Maximum permissible concentration)
NDSCh	Najwyższe Dopuszczalne Stężenie Chwilowe (Maximum Permissible Momentary Concentration)
NDSP	Najwyższe dopuszczalne stężenie pułapowe (Maximum permissible concentration threshold)
OSHA	Occupational Safety and Health Administration
CEIDARS	California Emissions Inventory Data Analysis and Reporting System.
PEL-TWA	Permissible Exposure Limit - Time-Weighted Average
REL-STEL	Recommended Exposure Limit - Short-Term Exposure Limit
IRIS RfC	Integrated Risk Information System Acute Reference Concentration.
crit. stab.clas	represents the critical atmospheric stability class at which pollutant concentrations reach their maximum value at the point of deposition
crit. wind. s.	represents the critical wind speed at which pollutant concentrations reach their maximum value at the point of deposition
crit. wind. d.	represents the critical wind direction at which pollutant concentrations reach their maximum value at the point of deposition

## References

[1] Najwyższa Izba Kontroli (2022). Zapobieganie Pożarom Miejsc Gromadzenia Odpadów.

[2] Li B., Mu Y. (2024). Impact of China’s National Sword Policy on Waste Import Margins: A Difference-in-Differences Approach. Sustainability.

[3] Minister Gospodarki (2015). Rozporządzenie Ministra Gospodarki z Dnia 16 Lipca 2015 r. w Sprawie Dopuszczania Odpadów do Składowania na Składowiskach.

[4] National Service Center for Environmental Publications (NSCEP) (2021). An Analysis of Lithium-Ion Battery Fires in Waste Management and Recycling.

[5] Winslow K.M., Laux S.J., Townsend T.G. (2018). A Review on the Growing Concern and Potential Management Strategies of Waste Lithium-Ion Batteries. Resour. Conserv. Recycl..

[6] Kersten M., Moor H.C., Johnson C.A. (1997). Speciation of trace metals in leachate from a MSWI bottom ash landfill. Appl. Geochem..

[7] Głodek E. (2011). Porównanie wielkości emisji zanieczyszczeń dla różnych opcji spalania odpadów. Pr. Inst. Ceram. Mater. Bud..

[8] Ryan J.V., Lutes C.C. (1993). Characterization of Emissions from the Simulated Open-Burning of Non-Metallic Automobile Shredder Residue.

[9] De Marini D.M., Lemieux P.M., Ryan J.V., Brooks L.R., Williams R.W. (1994). Mutagenicity and Chemical Analysis of Emissions from the Open Burning of Scrap Rubber Tires. Environ. Sci. Technol..

[10] Ministerstwo Środowiska (2010). Rozporządzenie Ministra Środowiska z Dnia 26 Stycznia 2010 r. w Sprawie Wartości Odniesienia dla Niektórych Substancji w Powietrzu.

[11] Trozzi C., Nielsen O.-K., Hjelgaard K., Wenborn M., Woodfield M. (2023). EMEP/EEA Air Pollutant Emission Inventory Guidebook 2023.

[12] Aurell J., Gullett B.K., Yamamoto D. (2012). Emissions from Open Burning of Simulated Military Waste from Forward Operating Bases. Environ. Sci. Technol..

[13] Seńczuk W. (2020). Toksykologia Współczesna.

[14] Vicente E.D., Figueiredo D., Goncalves C., Vicente A., Lopes I., Oliveira H., Alves C.A. (2025). PM10 exposure in beauty salons: Impact on A549 cell viability, cell cycle, oxidative stress, and mutagenicity. Environ. Toxicol. Pharmacol..

[15] Lovell D.P. (2000). Dose–response and threshold-mediated mechanisms in mutagenesis: Statistical models and study design. Mutat. Res. Genet. Toxicol. Environ. Mutagen..

[16] Adeniran J.A., Aremu A.S., Abdulraheem K.A. (2024). Modelling of air emissions from open burning of municipal waste in Ilorin Metropolis. Environ. Qual. Manag..

[17] U.S. Environmental Protection Agency (EPA) (1995). Stationary Point and Area Sources. AP-42: Compilation of Air Pollutant Emission Factors.

[18] Juda-Rezler K. (2006). Oddziaływanie Zanieczyszczeń Powietrza na Środowisko.

[19] Lönnermark A., Blomqvist P., Marklund S. (2008). Emissions from simulated deep-seated fires in domestic waste. Chemosphere.

[20] Ruokojärvi P., Ruuskanen J., Ettala M., Rahkonen P., Tarhanen J. (1995). Formation of polyaromatic hydrocarbons and polychlorinated organic compounds in municipal waste landfill fires. Chemosphere.

[21] Dominguez T., Aurell J., Gullett B., Eninger R., Yamamoto D. (2018). Characterizing emissions from open burning of military food waste and ration packaging compositions. J. Mater. Cycles Waste Manag..

[22] U.S. Environmental Protection Agency (EPA) (1992). Stationary Point and Area Sources. AP-42: Compilation of Air Pollutant Emission Factors.

[23] Bundesministerium für Umwelt, Naturschutz und nukleare Sicherheit (BMU) (2021). Erste Allgemeine Verwaltungsvorschrift zum Bundes-Immissionsschutzgesetz (Technische Anleitung zur Reinhaltung der Luft–TA Luft).

[24] Bundesrepublik Deutschland (1974). Bundes-Immissionsschutzgesetz (BImSchG)–Gesetz zum Schutz vor Schädlichen Umwelteinwirkungen durch Luftverunreinigungen, Geräusche, Erschütterungen und Ähnliche Vorgänge.

[25] U.S. Environmental Protection Agency (EPA) (2024). 40 CFR Part 51, Appendix W to Part 51—Guideline on Air Quality Models.

[26] Environment Agency (2021). Air Emissions Risk Assessment for Your Environmental Permit; Environmental Permitting Regulations Guidance.

[27] Pasquill F. (1979). Atmospheric Dispersion Modeling. J. Air Pollut. Control. Assoc..

[28] Minister Środowiska (2012). Rozporządzenie Ministra Środowiska z Dnia 24 Sierpnia 2012 r. w Sprawie Poziomów Niektórych Substancji w Powietrzu.

[29] The National Institute for Occupational Safety and Health (NIOSH) (2013). Current Intelligence Bulletin 66: Derivation of Immediately Dangerous to Life or Health (IDLH) Values.

[30] Ministerstwo Rodziny, Pracy i Polityki Społecznej (2018). Rozporządzenie Ministra Rodziny, Pracy i Polityki Społecznej z Dnia 12 Czerwca 2018 r. w Sprawie Najwyższych Dopuszczalnych Stężeń i Natężeń Czynników Szkodliwych dla Zdrowia w Środowisku Pracy.

